# Thermodynamic modelling of cements clinkering process as a tool for optimising the proportioning of raw meals containing alternative materials

**DOI:** 10.1038/s41598-023-44078-7

**Published:** 2023-10-16

**Authors:** Ana R. D. Costa, Mateus V. Coppe, Wagner V. Bielefeldt, Susan A. Bernal, Leon Black, Ana Paula Kirchheim, Jardel P. Gonçalves

**Affiliations:** 1 Polytechnic School, Post-Graduate Program in Civil Engineering (PPEC), Federal University of Bahia (UFBA), Salvador, 40210-630 Brazil; 2 School of Civil Engineering, University of Leeds, Leeds, LS2 9JT UK; 3 Iron and Steelmaking Laboratory (LASID), Department of Metallurgy, Post-Graduate Program in Mining, Metallurgical, and Materials Engineering (PPGE3M), Federal University of Rio Grande do Sul (UFRGS), Porto Alegre, 91501-970 Brazil; 4 Building Innovation Research Unit (NORIE), Department of Civil Engineering, Federal University of Rio Grande do Sul (UFRGS), Porto Alegre, 90035-190 Brazil; 5 Interdisciplinary Centre of Energy and Environment (CIENAM), Federal University of Bahia (UFBA), Salvador, 40170-115 Brazil

**Keywords:** Civil engineering, Sustainability, Materials chemistry, Environmental impact

## Abstract

The valorisation of waste or by-products in Portland clinker production is a promising alternative for developing sustainable cements. The complexity of the chemical reactions during clinkering demands an adequate dosing method that considers the effect of feedstock impurities to maximise the potential substitution of natural resources by waste or by-products, while guaranteeing the clinker reactivity requirements. This study proposes a raw meal proportioning methodology for optimising co-processing of natural feedstocks with alternative raw materials in clinker production, intending to reduce the content of natural raw materials needed, while promoting an optimal clinker reactivity. A thermodynamic modelling sequence was developed considering the variability of raw materials composition and heating temperatures. The model was then validated by comparing simulation outcomes with results reported in previous studies. An experimental case study was conducted for validation of the proposed method using a spent fluid catalytic cracking catalyst (SFCC), a by-product from the oil industry as an alternative alumina source during clinkering. The modelling simulations indicated that substitution of natural feedstocks by 15 wt% SFCC promotes the formation of reactive clinkers with more than 54% tricalcium silicate (C_3_S). Mixes with the potential to form the highest C_3_S were then produced, and heating microscopy fusibility testing was applied for evaluating the clinkers’ stability. The main factors governing the reactivity and stability of the clinker phases were the melt phase content, alumina modulus, and formation of C_3_S and dicalcium silicate (C_2_S). The self-pulverisation of clinker during cooling was observed in selected mixes, and it is potentially associated with high viscosity and low Fe content in the melt phase. The proposed framework enables optimisation of the dosing of raw meals containing alternative alumina-rich feedstocks for clinker production and allows a deeper interpretation of limited sets of empirical data.

## Introduction

World cement production is about 4.3 billion tons per year^1^, with an average consumption of raw materials of about 1.6 tonnes for each tonne of cement manufactured^2^. Valorisation of waste or by-products from different processes, as raw materials in cement kilns, is a common practice for reducing the environmental impacts associated with the extraction of natural resources, production costs, the volume of landfilled wastes, and the emission of greenhouse gases^3^. Furthermore, this approach has a sustainable benefit, as most of the potentially contaminating elements that might be present in alternative feedstocks are immobilised or solidified in the clinker^5^. On the other hand, minor elements can significantly compromise cement properties, demanding an adequate dosing method that considers the particularities of these industrial residues.

Bogue equations estimate the theoretical or potential composition of Portland clinker^5,6^. This method or its derivatives, combined with chemical moduli (alumina (AM), silica (SM), and lime saturation factor (LSF)), are the most frequently used for raw meal proportioning for research and industrial applications. The calculations assume the complete reaction of raw materials to form clinker compounds under equilibrium conditions and also ignore the possible effect of impurities^7^. However, the equations only give approximate clinker compositions and industrial clinkers show slight differences from the theoretical predictions. Furthermore, deviations can be even greater when impurities are present due to impacts on clinker phase stability, thus impacting the final properties of the cement^8^. Temperature variations, the composition of the kiln atmosphere, the presence of minor elements, and the absence of equilibrium conditions from cooling can all influence the development of the clinker phases, thus reducing the accuracy of the phase compositions estimated by Bogue's calculations^9,10^. Furthermore, minor elements within alternative raw materials or fuels influence the clinkering process^11^. The nature and quantity of these constituents can lead to notable changes in various aspects, including the morphology and proportion of the clinker phases^12^, the formation of minor phases^13^, the optimal clinkerisation temperature^14^, kiln residence time^15^, melt phase viscosity^16^, transformations on cooling^17^, the polymorphism of the main phases^18^, and the clinker reactivity^19^. In this scenario, thermodynamic modelling is an alternative tool to consider all these variables and to expand the scope of usable alternative materials.

Thermodynamic modelling enables development of simulations that account for the variability of the chemical composition of raw materials and conditions adopted during the heat treatment. By computing the effect of a wide range of trace oxides, the technique increases the accuracy of phase predictions, helping to solve problems associated with the synthesis of clinker prepared with raw materials containing impurities^10,20,21^. Early studies have focused on investigating systems containing the main clinker oxides (CaO, Fe_2_O_3_, Al_2_O_3_, and SiO_2_), expanding the understanding of clinker manufacturing. The studies utilised modelling techniques to explore the limitations of Bogue's predictions. The results demonstrated the absence of targeted compositions of clinker phases and lack of equilibrium during the cooling process, changing the proportion of the quantified phases^10^. Through the inclusion of minor elements in the clinker raw meal, the modelling accurately predicted the composition of the clinker produced on an industrial scale, allowing the evaluation of the effect of oxygen in the kiln atmosphere on the material quality^20^ and the concentration of impurities (alkalis, magnesium, zinc, chromium, and nickel) in the different kiln zones^22^. Additionally, a modelling methodology was employed to assess the impact of phosphorus on the formation of solid solutions of dicalcium and tricalcium silicate during clinkerisation^23^ and to predict the composition of belite clinker and calcium sulfoaluminate clinker containing blast furnace slag and red mud respectively as alternative raw materials^24,25^.

Previous investigations have also utilised modelling to examine the effect of minor oxides (SO_3_, Na_2_O, K_2_O, TiO_2_, and MgO) and mineralisers (CaF_2_, AlF_3_, MgSiF_6_, Na_2_SiF_6_, CaCl_2_, ZnO, and CaSO_4_) added to the clinker raw meal, reporting changes in phase formation during clinkerisation and the existence of an optimal mineraliser content^26^. Modelling the gradual replacement of Al_2_O_3_ by Fe_2_O_3_ has broadened the understanding regarding the effects of co-processing alternative iron-rich residues^27^. Hanein et al*.*^9^ developed an optimised thermodynamic dataset for clinker phases to overcome the limitations of databases not freely available or primarily designed for other applications. Although thermodynamic modelling has allowed advances in the clinkering process and predicting the impact of minor elements, its potential for optimising the dosing process of raw clinker meal remains unexplored. This application can enhance the understanding of the effect of combined minor elements and enable the maximisation of co-processing alternative raw materials in cement kilns, ultimately producing more sustainable clinkers.

One of the biggest challenges in cement manufacture at an industrial scale is maintaining the consistency of the clinker phases over multiple production cycles. Such variability stems from using alternative feedstocks such as waste or by-products, containing impurities, which can induce temperature changes inside the kiln and during the cooling process^8^. Different minor impurities can stabilise or destabilise specific silicate phases over others, induce the formation of new clinker phases, and compromise the reactivity and stability of the cement when hydrated^28–31^. Predictive modelling tools can mitigate all these factors. It allows for the identification of optimised raw meal formulations shortening experimental programs, optimising the dosing of raw meals containing alternative feedstocks, and enabling a broader interpretation of limited sets of empirical data.

Aluminous residues have been a relevant alternative raw material in clinker production^32–39^. They allow for the reduction of the environmental impacts associated with the extraction of natural clays, such as deforestation, soil surface destruction or degradation, slope instability, erosion, and siltation^40^. Spent fluid catalytic cracking catalyst (SFCC) is a residue of the fluidised bed catalytic cracking process in oil refining units^41^. Due to the high amounts of Al_2_O_3_ and SiO_2_ (generally totalling more than 80%), SFCC is classified as a potential partial substitute raw material in cement manufacture^42–45^. SFCC has been co-processed in clinker manufacture to reduce production costs and energy, but its replacement content in raw meals was limited to 4 wt%^30,46,47^.

The composition of complementary raw materials, chemical moduli, and the target composition of the clinker influence the content of co-processed SFCC. However, the reduced reactivity and compressive strength of clinker containing more than 4 wt% of SFCC were attributed to the effect of impurities in the waste material and the formation of high levels of tricalcium aluminate^30^. Maximising the C_3_S content could enhance the reactivity and compressive strength of the system produced with SFCC. In this sense, applying a raw meal proportioning method using thermodynamic modelling allows consideration of the influence of minor elements on the clinker phases, melt phase content, clinker stability, transition phases, and potential cement reactivity. It is thus possible to maximise the recyclable SFCC content in the clinker while guaranteeing the reactivity requirements, proper waste disposal, and reducing the cost of using and exploiting natural raw materials.

In this study, a raw meal proportioning method is proposed for the optimisation of raw meal formulation during the co-processing of natural and alternative feedstocks for clinker production. The method comprises thermodynamic modelling and heating microscopy as tools for system simulation and decision-making. The analysis aims to maximise the content of co-processed waste material while ensuring maximum reactivity of the cement produced. Then, this research presents a case study using the SFCC with the mixing dosage based on the proposed methodology. A final method is presented for the application of any alumina-rich waste material in clinker production.

## Experimental methodology

### Materials

In this study, SFCC and analytical purity reagents were used: silicon oxide (SiO_2_; CAS 60676-86-0), iron III oxide (Fe_2_O_3_; CAS 1309-37-1), aluminium oxide (Al_2_O_3_; CAS 1344-28-1), and calcium carbonate (CaCO_3_; CAS 471-34-1). Analytical purity (AP) materials were applied to isolate the effects of SFCC impurities and potentially contaminating elements. The oxide composition of the raw materials used (Table 1) was obtained by X-ray fluorescence spectrometry (XRF) using an S8 Tiger (Bruker) instrument.

**Table 1:** Oxide composition of the raw materials (wt%).

Oxides (wt%)	CaCO_3_ AP	SiO_2_ AP	Fe_2_O_3_ AP	Al_2_O_3_ AP	SFCC
CaO	54.95	0.01	0.02	0.07	0.10
SiO_2_	0.17	89.56	0.17	0.19	47.97
Fe_2_O_3_	0.02	0.05	97.53	0.02	1.13
Al_2_O_3_	0.02	0.51	0.08	95.26	42.09
MgO	0.29	n.d	n.d	n.d	0.03
SO_3_	0.02	0.26	0.49	0.02	0.10
P_2_O_5_	0.07	n.d	0.02	19 ppm	0.41
Na_2_O	0.02	0.56	0.10	0.36	0.51
ZnO	16 ppm	9 ppm	0.04	38 ppm	0.01
K_2_O	61 ppm	n.d	20 ppm	19 ppm	0.15
NiO	17 ppm	30 ppm	0.01	19 ppm	0.89
TiO_2_	17 ppm	0.02	59 ppm	48 ppm	0.52
MnO	56 ppm	n.d	0.18	n.d	39 ppm
La_2_O_3_	n.d	n.d	39 ppm	n.d	2.39
V_2_O_5_	6 ppm	n.d	20 ppm	48 ppm	0.71
Others	0.30	0.01	0.18	0.07	0.22
LOI	44.14	9.02	1.17	4.01	2.77

*LOI* Loss on ignition (1000 °C), as determined by thermal analysis; *n.d.* Not detected; *ppm* Parts per million.

SFCC is an industrial residue collected from an oil refinery in Bahia, Brazil. Silicon, aluminium, and iron oxides account for more than 91 wt% of the material. Considering its oxide composition (Table 1), the SFCC has an Al_2_O_3_/SiO_2_ ratio of 0.88. This ratio is close to the lower limit reported for SFCC from different sources, which varies between 0.65^48^ and 1.70^30^. SFCC generated in South America generally has values below 0.9, characteristic of catalysts with higher thermal stability^49–51^. The catalyst prior to use is a zeolite (aluminosilicate), a microporous crystalline solid^52,53^. The sample contains 2.39 wt% lanthanum oxide. This element is incorporated into the zeolite structure, increasing catalytic reactivity, hydrothermal stability, and life expectancy during catalytic cracking^54^. La-modified zeolite Y is usually manufactured from the conversion of NaY zeolites, justifying the Na_2_O_3_ content in the sample^55^. The most relevant minor elements in SFCC are transition metals (Fe, Ni, V, and Ti), and P. Phosphorus is potentially absorbed by zeolite LaY^56^. Transition metals come from crude oil^57,58^.

Colloidal silicon dioxide (SiO_2_ AP) showed a high LOI (9%), as this material has a high moisture adsorption capacity^59^. Although storage conditions were guaranteed, the sample adsorbed moisture during handling. The LOI determined by thermogravimetric analysis (TG) indicated that the mass loss occurs at temperatures below 100 °C, being attributed to the presence of unbound water. The same was observed for Al_2_O_3_ AP. The mass losses below 100 °C were ignored in thermodynamic modelling calculations since the raw meals were dried in an oven at this temperature before clinkering.

**Figure 1:**
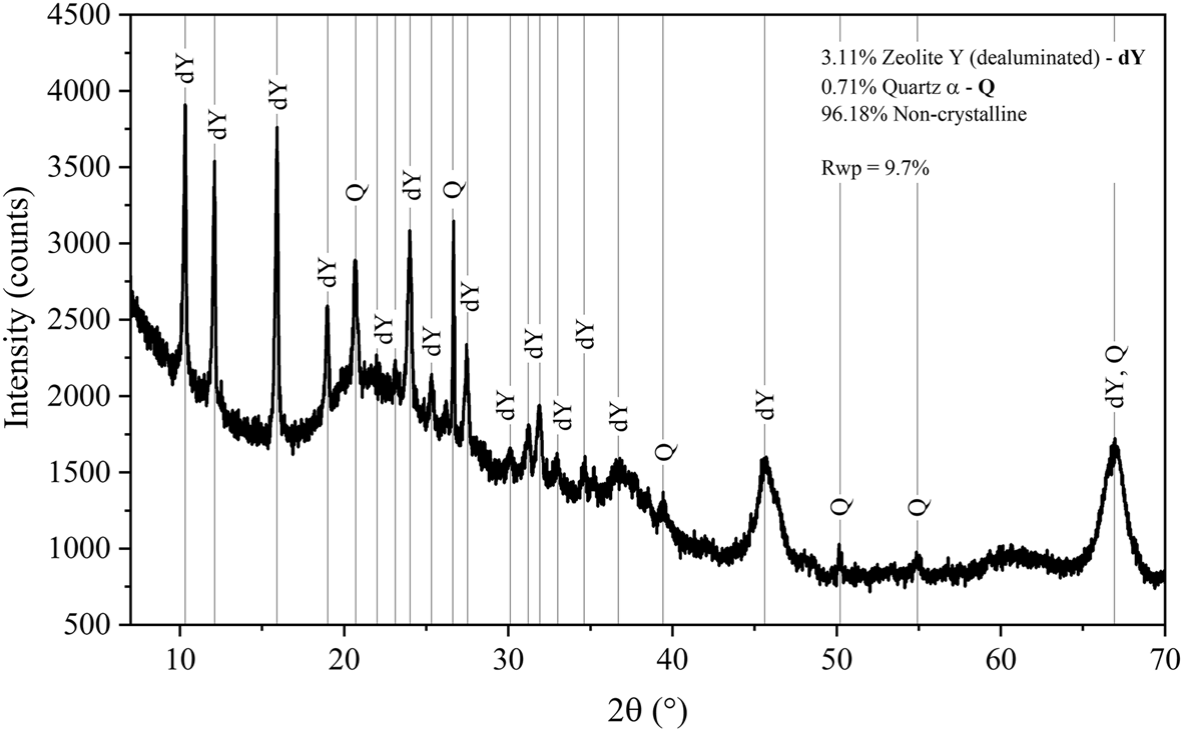
X-ray diffractogram and mineral composition of SFCC obtained by XRD/Rietveld. dY: dealuminated zeolite Y (ICSD 41395, Al_1.72_(Al_0.48_Si_9.84_O_22.98_)); Q: quartz (ICSD 83849, SiO_2_).

Figure 1 shows the phase composition of the SFCC quantified by X-ray diffractometry combined with Rietveld refinement (XRD/Rietveld). The amorphous content was determined by the internal standard method, using 20 wt% corundum (Al_2_O_3_; CAS 1344-28-1). The sample is composed of type Y dealuminated zeolite and α-quartz. No peaks attributed to lanthanum oxide or other La-based phases were detected. This suggests that La is incorporated into the Y zeolite structure or highly dispersed on the zeolite surface^52,55^. The main types of zeolites used in catalytic cracking are X, Y, and ZSM-5^54^. Zeolite Y has the higher stability. The XRD pattern shows an amorphous halo verified by the baseline non-linearity. The sample has a high amorphous content (96.18%). This is attributed to the hydrothermal dealumination during catalytic cracking, partially decomposing the crystalline structure of the zeolite^58^.

### Methods

#### Raw meal proportioning by thermodynamic modelling

The proposed dosing method calculates the raw meal composition aiming to include SFCC as an alumina source and maximising the C_3_S content. The modelled systems were designed to use up to 20 wt% SFCC in the raw meal, which was proportioned using FactSage version 7.3^60^. The software is a thermodynamic tool used for clinker equilibrium calculations in previous investigations^23,26,27,61^. The simulations used thermodynamic databases for gaseous components (FactPS) and oxides in solid, liquid (melt), and solution phases (FToxid)^23,60^.

The modelling approach adopted was applied following the subsequent considerations: (1) The number of oxides considered for each system was limited to 14 due to the data processing time. Processing times for models with more oxides exceeded 24 h; (2) The initial step considered clinkering up to 1450 °C; (3) The modelling considered a 0.5% step size in the content of each raw material for each scenario; (4) The content range for each raw material was delimited considering the usual values for clinkerisation of a CEM I (cement with a maximum of 5% other constituents)^5^. In this sense, the CaCO_3_ AP content varied from 75 to 100% of the non-SFCC fraction in the raw meal. For SiO_2_ AP and Fe_2_O_3_ AP, the applied ranges were 0 to 25%; (5) The SFCC content (5, 10, 15, and 20%) was fixed for each system; (6) 2601 scenarios (raw meal compositions) were modelled; (7) The optimal blend of raw materials was defined as the scenario giving maximum C_3_S formation at 1450 °C.

As input data, the proportions of the raw materials were varied using the oxide compositions and equations in linear systems. The oxide contents of each raw material were normalised to 100%, considering the contents of Al_2_O_3_, CaO, CO_2_, Fe_2_O_3_, K_2_O, MgO, MnO, Na_2_O, NiO, P_2_O_5_, SiO_2_, SO_3_, TiO_2_, and ZnO. The CO_2_ content was calculated by the mass loss between 500 and 1000 °C upon thermogravimetric analysis. The system pressure was set at 1 atm. The clinkering was set at 1450 °C, a temperature at which the Lime Saturation Factor (LSF) is established, and the reaction of CaO to form C_3_S is optimised^5,6^. Furthermore, this is a typical temperature for ordinary cement clinker production^5^. However, this value can be adjusted according to the intended application.

The FactSage software allows the insertion of first-degree equations for each input oxide, enabling the modelling of several systems in an automated way as a function of a common variable. The proposed dosing method uses this tool combined with a set of linear systems, aiming to cover a range of possible scenarios when varying the proportioning of raw materials.

The software calculation consisted of three main steps. For each oxide, it was necessary to define a linear equation to simulate all scenarios. The independent variable (X) was the SiO_2_ AP content, and the dependent variable (Y) was the oxide content in the scenario. The angular coefficient (A) was calculated according to Eq. (1). Where Ai is the slope for each oxide, n is the total number of oxides considered in the system, and a_i,j_ is the content of each oxide, with i referring to the oxide and j referring to the raw material.

1 $$\left[ {\begin{array}{*{20}l} {{\text{A}}_{1} } \\ {{\text{A}}_{2} } \\ \ldots \\ {{\text{A}}_{{\text{n}}} } \\ \end{array} } \right] = \left[ {\begin{array}{*{20}l} {{\text{a}}_{{1,1}} } & {{\text{a}}_{{1,2~}} } & {{\text{a}}_{{1,3}} ~} & {{\text{a}}_{{1,4}} } \\ {{\text{a}}_{{2,1}} } & {{\text{a}}_{{2,2~}} } & {{\text{a}}_{{2,3}} } & {{\text{a}}_{{2,4}} } \\ \ldots & \ldots & \ldots & \ldots \\ {{\text{a}}_{{{\text{n}},1}} } & {{\text{a}}_{{{\text{n}},2~}} } & {{\text{a}}_{{{\text{n}},3}} } & {{\text{a}}_{{{\text{n}},4}} } \\ \end{array} } \right] \cdot \left[ {\begin{array}{*{20}l} 0 \\ { - 1} \\ 1 \\ 0 \\ \end{array} } \right]$$

The linear coefficient (B) for each oxide was calculated from Eq. (2). Where Sfixed was the fixed SFCC content for the system, Cmax was the maximum CaCO_3_ AP content in the raw meal (100–Sfixed–Ffixed), Smin was the minimum content of SiO_2_ AP (0%), and Ffixed was the content of Fe_2_O_3_ AP fixed in the system (varying in 0.5% increments from 0 to 25%).

2 $$\left[ {\begin{array}{*{20}l} {{\text{B}}_{1} } \\ {{\text{B}}_{2} } \\ \ldots \\ {{\text{B}}_{{\text{n}}} } \\ \end{array} } \right] = \left[ {\begin{array}{*{20}l} {{\text{a}}_{{1,1}} ~} & {{\text{a}}_{{1,2~}} } & {{\text{a}}_{{1,3}} } & {{\text{a}}_{{1,4}} } \\ {{\text{a}}_{{2,1}} } & {{\text{a}}_{{2,2~}} } & {{\text{a}}_{{2,3}} } & {{\text{a}}_{{2,4}} } \\ \ldots & \ldots & \ldots & \ldots \\ {{\text{a}}_{{{\text{n}},1}} } & {{\text{a}}_{{{\text{n}},2~}} } & {{\text{a}}_{{{\text{n}},3}} ~} & {{\text{a}}_{{{\text{n}},4}} } \\ \end{array} } \right] \cdot \left[ {\begin{array}{*{20}l} {{\text{S}}_{{{\text{fixed}}}} } \\ {{\text{C}}_{{\max }} } \\ {{\text{S}}_{{\min }} } \\ {{\text{F}}_{{{\text{fixed}}}} } \\ \end{array} } \right]$$

Up to this point, the method allowed modelling the clinkering of raw meals with the alumina modulus (AM), Al_2_O_3_/Fe_2_O_3_ mass ratio, allowed to vary freely. Samples dosed by this method were named S5, S10, S15, and S20, according to the SFCC content. The amount of SFCC was fixed to streamline the equations and improve calculation times. No additional alumina source was introduced, and optimisation was based on the predicted maximum C_3_S content. Consequently, the dosage of complementary raw materials varied among the different systems. As the SFCC content increased, the availability of CaO for C_3_S formation decreased. As a result, each amount of co-processed SFCC has a maximum potential for C_3_S formation. In this sense, the conventional approach of establishing a target clinker composition and dosing the raw meal by continuously varying the alternative material content would restrict the co-processing potential of the SFCC. This constraint would limit the predicted C_3_S content and, consequently, the reactivity of the resulting clinker. For this reason, the quantity of SFCC was fixed for simplicity, and the discussion focused on optimised dosages. However, the proposed method maps the predicted C_3_S content for all possible combinations, enabling the identification of a specific target composition in all cases.

Due to the high alumina content of SFCC, the calculation process to maximise C_3_S content tended to reduce Fe_2_O_3_ in the raw meal, and consequently the AM tended to exceed typical values seen in industrial clinker production of 1.6^5^. In the initial method, its value tended to exceed 25. Therefore, a second step was added, fixing the AM value at 1.6. The determination of the Ffixed term (Fe_2_O_3_ AP content) used in Eq. (2) was now calculated from Eq. (3). After this method adaptation, the samples dosed were named S5F, S10F, S15F, and S20F, where F indicates the AM fixed at 1.6. An applied example of the method equations is presented in the Supplementary File.

3 $${\text{F}}_{{{\text{fixed}}}} = \frac{{{\text{S}}_{{{\text{fixed}}}} \cdot \% {\text{Al}}_{2} {\text{O}}_{{3\,{\text{SFCC}}}} }}{{{\text{AM}} \cdot \% {\text{Fe}}_{2} {\text{O}}_{{3\,{\text{Fe}}_{2} {\text{O}}_{3} \,{\text{AP}}}} }}$$

The computational modelling was processed, resulting in the clinker phases in equilibrium at 1450 °C. For each SFCC content, 2601 scenarios were modelled, totalling 20,808 results. For the next step of each system, the scenario with the maximum C_3_S content was chosen. The CO_2_ emissions from limestone decarbonation were calculated based on the optimised composition of the raw meals. The calculation assumed that all the calcium in each material is present as CaCO_3_ and considered the data from Table 1 to determine the amount of clinker produced for each tonne of raw materials.

After the raw meal proportioning, the phases of the systems were modelled by simulating the heat treatment in industrial production. The processing was applied using the FactSage equilibrium module and adopting the entire set of outputs available in the system. The equilibrium composition calculation was applied between 1000 and 1450 °C with 10 °C steps. The cooling simulation was performed using the Scheil-Gulliver model^23^. In this case, the temperature and phases obtained after the maximum conversion of the melt phase (formed at the maximum clinkering temperature) into solids were determined during system cooling (starting from 1450 °C). The melt phase remaining after cooling corresponds to the chemical composition of the non-crystalline phases of clinker^62^.

Thermodynamic modelling through FactSage considers the rhombohedral structure of tricalcium silicate (Ca_3_SiO_5_)^23^. For the dicalcium silicate (Ca_2_SiO_4_), the software presents the gamma, alpha prime, and alpha polymorphs represented by the indices s1, s2, and s3^60^. In addition to the solids, the simulations detail the chemical composition of the molten and gaseous phases. The main phases within clinker are commonly reported as solid solutions due to the inclusion of minor elements within their crystalline structures^5,63,66^. The triclinic and monoclinic polymorphs of Ca_3_SiO_5_ (T1, T2, T3, M1, M2, and M3) are distortions of its rhombohedral phase^5^. Similarly, beta Ca_2_SiO_4_ represents an impure form of dicalcium silicate, as pure β-C_2_S is unstable under normal atmospheric conditions^65^. Although the calculations presented in this study did not include solid solutions of silicates, the modelling considered new phases resulting from the incorporation of minor elements (Mg_2_SiO_4_, Ni_2_SiO_4_, Zn_2_SiO_4_, Ca_2_MnO_4_, Ca_3_Ti_2_O_6_, and others^60^), in addition to solid solutions of aluminate phases. Portland cement clinker typically contains aluminium-bearing phases, such as tricalcium aluminate (C_3_A) and calcium aluminium ferrite (C_4_AF). C_3_A (Ca_3_Al_2_O_6_) has a cubic structure but can incorporate alkali metals and be converted into an orthorhombic form^66^. In this sense, the incorporation of sodium can result in the formation of Na_2_Ca_8_Al_6_O_18_^67,68^. C_4_AF has the formula Ca_2_(Al_x_Fe_1-x_)_2_O_5_, with the Al/Fe ratio varying according to raw meal composition and clinkering conditions^63^. It can also contain up to 10% of other constituents based on Ca, Al, Fe, and O_5_. Ca_2_Fe_2_O_5_ contains Fe^3+^ in octahedral and tetrahedral sites. As the Al content is increased, it preferentially occupies the tetrahedral and later the octahedral sites, modifying the crystalline structure^5^. Furthermore, C_4_AF frequently exhibits variable composition zones due to fractionation during cooling^9^. Thermodynamic modelling using FactSage considers the formation of solid solutions of calcium aluminium ferrites, including Ca(Al,Fe)_2_O_4_, Ca_2_(Al,Fe)_2_O_5_, and Ca_3_(Al,Fe)_2_O_6_^60^. Where (Al,Fe) means Al and Fe are interchangeable in the structure. Therefore, the quantification of Ca_3_(Al,Fe)_2_O_6_ in FactSage contains the C_3_A amount, and the complementary fraction is attributed to intermediate phases in forming C_4_AF. In this context, previous studies discussed the C_3_A and C_4_AF content as the general sum of the CaO-Al_2_O_3_-Fe_2_O_3_ solid solutions mentioned and named as C-A-F^9,20,23^. In this study, the solutions are presented individually (C(A,F), C_2_(A,F), and C_3_(A,F)) to provide greater clarity about the phase changes according to the raw meal composition.

#### Fusibility test by heating microscopy

Heating microscopy was used to define the compositions with the highest production potential on an industrial scale. As eliminatory criteria, melting at temperatures below 1450 °C or collapse after cooling were considered. The fusibility test was performed using a 1600 heating microscope (LEITZ). The raw meal samples (Table 2) were shaped into cylindrical specimens (Ø 2 mm × 3 mm) and placed on an alumina support in the heating unit. The samples were heated from room temperature with a heating rate of 12 °C/min up to 800 °C, followed by 10 °C/min up to 1450 °C. The specimens were cooled at a rate of 35 °C/min from 1450 °C down to 200 °C at 1 atm. The varying heating rates were used following DIN 51730^69^.

**Table 2:** Detailed composition (wt%) and parameters of the clinker raw meals produced for the fusibility test.

Sample	SFCC	CaCO_3_ AP	SiO_2_ AP	Fe_2_O_3_ AP	Al_2_O_3_ AP	LSF	SM	AM
S5	5.00	81.50	13.50	0.00	0.00	97	6.6	27.4
S7.5	7.50	81.00	11.50	0.00	0.00	97	4.3	30.2
S10	10.00	80.50	9.50	0.00	0.00	98	3.1	31.8
S15	15.00	79.00	6.00	0.00	0.00	96	2.0	33.6
S20	20.00	78.00	2.00	0.00	0.00	97	1.3	34.6
R5	0.00	81.59	16.11	0.06	2.24	97	6.6	26.5
R7.5	0.00	81.09	15.41	0.11	3.39	97	4.2	24.9
R10	0.00	80.68	14.71	0.12	4.49	98	3.1	31.1
R15	0.00	79.25	13.82	0.18	6.75	96	2.0	33.0
R20	0.00	78.34	12.41	0.24	9.01	97	1.3	34.1
S5F	5.00	81.00	12.62	1.38	0.00	99	4.0	1.6
S7.5F	7.50	80.00	10.42	2.08	0.00	100	2.5	1.6
S10F	10.00	79.00	8.23	2.77	0.00	100	1.8	1.6
S15F	15.00	77.00	3.85	4.15	0.00	100	1.0	1.6
S20F	20.00	74.46	0.00	5.54	0.00	97	0.7	1.6
R5F	0.00	81.06	15.23	1.46	2.26	99	4.0	1.6
R7.5F	0.00	80.09	14.33	2.19	3.39	99	2.5	1.6
R10F	0.00	79.12	13.44	2.92	4.52	100	1.8	1.6
R15F	0.00	77.17	11.67	4.38	6.78	100	1.0	1.6
R20F	0.00	74.69	10.43	5.84	9.04	96	0.7	1.6

*LSF* Lime saturation factor; S*M* Silica modulus (SiO_2_/(Al_2_O_3_ + Fe_2_O_3_); *AM* Alumina modulus (Al_2_O_3_/Fe_2_O_3_).

For heating microscopy, samples were selected based on the dosage method, specifically targeting those with maximum C_3_S formation. These selected samples were labelled as Si or SiF, with the letter "i" denoting the co-processed SFCC content and the letter "F" indicating that the AM was set at 1.6. Reference samples were calculated according to the chemical composition of the raw meal defined in the modelling for systems containing SFCC. In other words, for each sample with SFCC, a corresponding reference raw meal containing CaCO_3_ AP, SiO_2_ AP, Al_2_O_3_ AP, and Fe_2_O_3_ AP was dosed. The proportioning applied the least squares method, aiming to isolate the effect of potentially contaminating elements of the SFCC. The contents of the main oxides (CaO, SiO_2_, Al_2_O_3_, and Fe_2_O_3_) in the reference and SFCC samples were then approximated, and the SFCC minor elements highlighted. Additional samples of clinker raw meals containing 7.5% SFCC and their respective references were produced for the fusibility test. The oxide composition of the raw meals is detailed in the Supplementary File.

#### Framework validation

The results, parameters, and trends observed in the dosing method were validated based on 21 clinker samples synthesised in eight previous studies^23,31,70–75^. The validation consisted of simulating the clinkers produced by these authors and comparing the results with the trends observed in this study. Thermodynamic modelling followed the methodology presented in the raw meal proportioning method. The publications were chosen based on the following criteria: It presented the raw meal composition by XRF and thermogravimetric analysis (TG); it detailed temperature conditions and heating rate during clinkering; it was published in a peer-reviewed journal; it addressed the synthesis of Portland clinker or self-pulverising clinker based on silicates. Detailed raw meal composition and clinkering conditions are presented in the Supplementary File.

## Results and discussions

### Maximisation of C_3_S content

**Figure 2:**
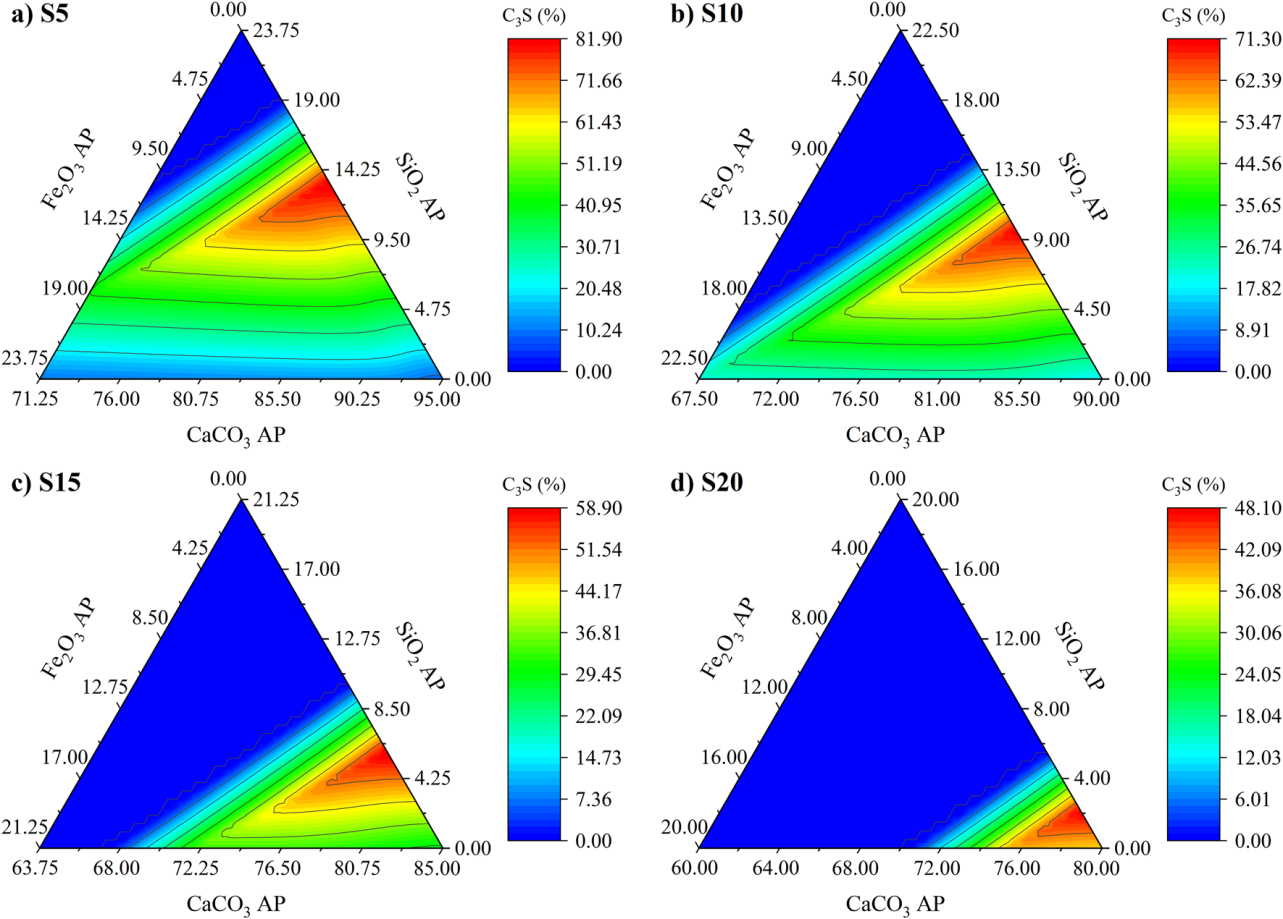
Thermodynamic modelling simulation outcomes showing composition ranges of the raw meal proportioning as a function of C_3_S formation after clinkering at 1450 °C. Simulations are shown considering the SFCC content in the raw meal where (**a**) 5% SFCC, (**b**) 10% SFCC, (**c**) 15% SFCC, and (**d**) 20% SFCC, respectively. AP stands for analytical purity reagent.

Tricalcium silicate (C_3_S or Ca_3_SiO_5_) is the most important phase of ordinary Portland cement (OPC), corresponding to between 50 and 70% of the material^63^. C_3_S has high hydraulic reactivity and is mainly responsible for compressive strength development up to 28 days of hydration^5^. Previous studies incorporating 8% SFCC as a residual raw material in clinker production, showed about a 30% reduction in compressive strength than OPC paste^30^. Qualitative XRD analysis performed by the authors also indicated that the intensity of the peaks attributed to C_3_S in the reference samples was approximately double that of the SFCC clinker^30^. Although the potential reactivity of clinker is influenced by the types of materials used in the raw meal, an appropriate dosing method can optimise the clinker composition regardless of the raw material sources. This is possible since thermodynamic modelling considers the effect of minor elements of all raw materials. While previous investigations on SFCC co-processing employed natural raw materials, the present study focuses on dosing with laboratory-grade raw materials to isolate the effects of SFCC impurities. In this context, the first step of the dosing method proposed in this study sought to maximise the C_3_S content, aiming to optimise the reactivity and compressive strength of the material. Figure 2 presents the C_3_S contents calculated by thermodynamic modelling. The diagrams are pseudo-ternary because they show the three non-SFCC raw materials. Thus, the sum is 100% when adding the SFCC content to the raw meal.

Increasing the SFCC content reduces the C_3_S content because the residue is an aluminosilicate and lacks Ca available to form silicates. However, the method maps the possible combinations of supplementary raw materials and indicates the point of maximum C_3_S formation. It is noteworthy that the dosing optimisation allows the production of clinkers containing more C_3_S (81.9%) compared to previous research, which reached 67.2% and 52.5% when co-processing 3.5%^46^ and 4.0%^30^ SFCC, respectively.

Table 3 presents the raw meal composition for the maximum C_3_S formation scenarios. Optimisation predicts a clinker with approximately 50% C_3_S even when 20% SFCC is co-processed in the raw meal. The Lime Saturation Factor (LSF) and Silica Modulus (SM) tend towards the usual limits for OPC production, which were 93–98 and 2.2–3.3, respectively^5^. The additional iron oxide reagent (Fe_2_O_3_ AP) was optimised to zero as the system maximised the CaCO_3_ content to reach sufficient calcium for C_3_S development. However, the system still contains iron, arising from the SFCC, which contributes to forming C_4_AF. This minimisation of iron results in maximising the alumina modulus (AM, Al_2_O_3_/Fe_2_O_3_ ratio).

**Table 3:** Raw materials proportioning (wt%), raw meal parameters, and CO_2_ emissions through decarbonation for the productions of clinker containing SFCC optimised by thermodynamic modelling.

Sample	SFCC	CaCO_3_ AP	SiO_2_ AP	Fe_2_O_3_ AP	C_3_S max	LSF	SM	AM	CO_2_ emissions (kg/t of clinker)
S5	5.00	81.50	13.50	0.00	82%	97	6.6	27.4	543.0
S10	10.00	80.50	9.50	0.00	71%	98	3.1	31.8	532.9
S15	15.00	79.00	6.00	0.00	59%	96	2.0	33.6	517.9
S20	20.00	78.00	2.00	0.00	48%	97	1.3	34.6	508.1

*LSF* Lime saturation factor; *SM* Silica modulus; *AM* Alumina modulus.

**Figure 3:**
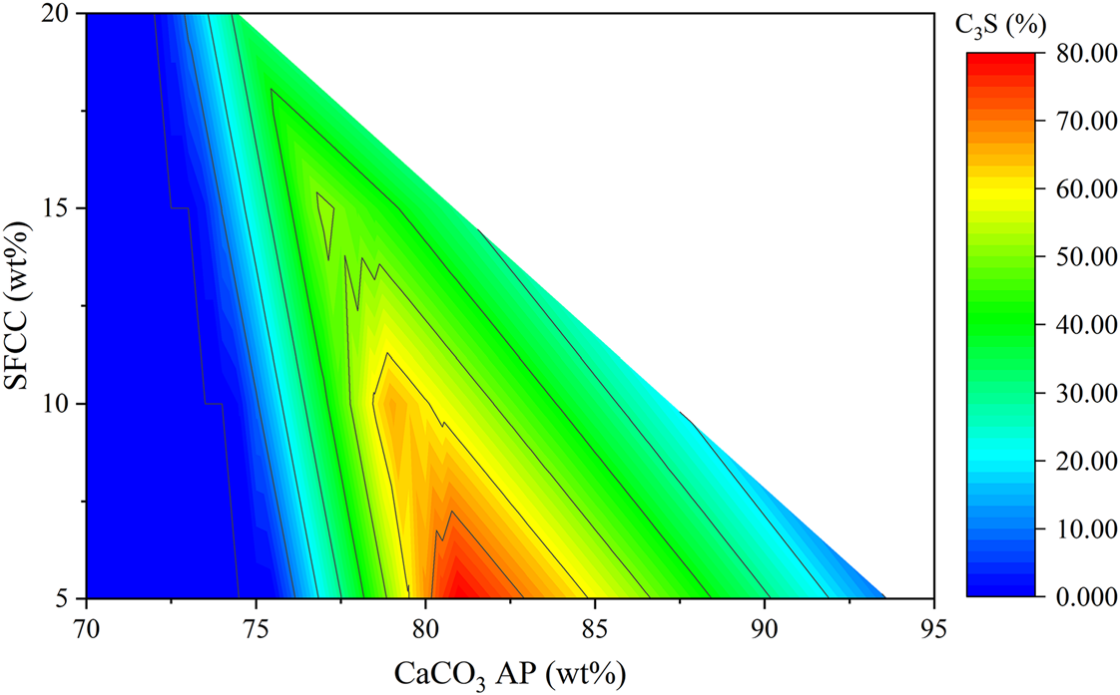
Diagram to identify the raw meal proportioning with maximum C_3_S formation after clinkering at 1450 °C and alumina modulus fixed at 1.6.

AM is related to the C_3_A/C_4_AF ratio in the clinker^5^. A high AM increases the melt phase viscosity during clinkering, delaying silicate conversion and C_3_S formation^76^, while increasing the iron content in the raw meal allows lower clinkering temperatures, optimising the C_3_S, and limiting the dicalcium silicate (Ca_2_SiO_4_) content^77^. Lin et al*.*^30^ related the poor reactivity of clinker prepared with SFCC to the high tricalcium aluminate content (19%). It is noteworthy that the type of raw materials used in the study may have influenced the stability of C_3_A rather than other phases. However, the elevated AM (3.5) may have contributed to maximising the C_3_A content in the clinker. Thus, the proposed dosing method was adapted by setting the AM at 1.6 to regulate the C_3_A content and optimise the conditions for C_3_S formation. The value was defined based on the usual range for OPC, which varies between 1.4 and 2.2^5^. Figure 3 shows the diagram corresponding to systems with the AM set to 1.6. As the SFCC is the only source of alumina, the Fe_2_O_3_ AP content was fixed for each residue content. The C_3_S formation zone narrowed as the SFCC content increased, as a consequence of CaCO_3_ depletion in the system. As a result, the limit of partial replacement of SFCC in raw meals is defined by the minimum C_3_S content required for the application. For example, for Portland clinker with at least 50% C_3_S, the co-processed SFCC content could reach up to 15% (Table 4).

**Table 4:** Raw materials proportioning (wt%), raw meal parameters, and CO_2_ emissions through decarbonation for the production of clinker containing SFCC optimised by thermodynamic modelling and fixing the AM at 1.6.

Sample	SFCC	CaCO_3_ AP	SiO_2_ AP	Fe_2_O_3_ AP	C_3_S max	LSF	SM	AM	CO_2_ emissions (kg/t of clinker)
S5F	5.00	81.00	12.62	1.38	80%	99	4.0	1.6	537.9
S10F	10.00	79.00	8.23	2.77	66%	100	1.8	1.6	517.8
S15F	15.00	77.00	3.85	4.15	52%	100	1.0	1.6	498.3
S20F	20.00	74.46	0.00	5.54	33%	97	0.7	1.6	474.2

*LSF* Lime saturation factor; *SM* Silica modulus; *AM* Alumina modulus.

Table 4 presents the composition of the raw meals with maximised C_3_S contents and AM fixed at 1.6. The chemical moduli are adequate for the recommended ranges for OPC^5^. Thermodynamic modelling enabled dosing more sustainable clinkers, optimising the composition of the raw meal, enhancing C_3_S, and reducing the CO_2_ emitted by the raw materials. Al-Dhamri and Melghit^46^ co-processed 3.5% SFCC and 86.8% limestone to produce a clinker with 67.2% C_3_S. Lin et al*.*^30^ used 4% SFCC and 78.0% CaCO_3_ and obtained 52.5% C_3_S. Limestone is the main raw material responsible for CO_2_ emissions during the clinkering process, volatilising about 44% of its mass as this pollutant^5,78^. In this sense, the dosed samples are potentially more reactive (higher in C_3_S) and consume less CaCO_3_, thus decreasing CO_2_ emissions.

Table 5 shows the composition of the clinkers after the cooling simulation, showing both those with AM varying freely (> 27), and those with AM fixed at 1.6. The C_3_S content is slightly higher than in Tables 3 and 4, as the Scheil-Gulliver cooling simulation demonstrates that the lack of equilibrium promoted by rapid cooling allows for more C_3_S to be retained^10^. The successive steps of the cooling calculation consider exclusively the equilibrium between the remaining melt and the solids formed from its solidification, progressively modifying the melt phase composition until reaching a eutectic point^10^.

**Table 5:** Predicted clinker composition at clinkering up to 1450 °C followed by rapid cooling (Scheil-Gulliver method). The number on the sample label denotes the co-processed SFCC content. The letter F or its absence indicates whether the AM was set at 1.6 or varied freely, respectively.

Composition (wt%)	S5	S10	S15	S20	S5F	S10F	S15F	S20F
Ca_3_SiO_5_	82.62	72.77	60.32	50.22	81.43	67.84	54.25	35.22
Ca_2_SiO_4_ α’	6.16	8.64	12.52	14.76	4.85	6.49	8.30	10.85
Ca_2_SiO_4_ α	1.46	0.00	1.04	0.00	0.00	0.00	0.00	5.71
Ca(Al,Fe)_2_O_4_	1.04	1.65	2.85	4.15	0.37	0.37	0.35	0.36
Ca_2_(Al,Fe)_2_O_5_	0.19	0.01	0.00	0.00	6.14	10.90	15.59	20.33
Ca_3_(Al,Fe)_2_O_6_	2.36	10.43	17.73	24.41	4.12	10.34	16.38	22.11
Ca_3_MgAl_4_O_10_	1.43	3.38	3.72	3.66	1.14	1.97	2.72	3.32
Na_2_Ca_8_Al_6_O_18_	3.16	0.86	0.00	0.00	0.00	0.00	0.00	0.00
Na_2_Ca_3_Al_16_O_28_	0.00	0.00	0.00	0.00	0.04	0.04	0.02	0.00
CaO	0.00	0.64	0.00	0.67	0.66	0.57	0.54	0.00
MgO	0.23	0.04	0.00	0.00	0.26	0.16	0.08	0.00
NiO	0.02	0.09	0.13	0.19	0.12	0.16	0.24	0.30
Ca_3_Ti_2_O_7_	0.06	0.15	0.25	0.33	0.07	0.16	0.25	0.32
Melt	1.26	1.33	1.45	1.62	0.78	0.97	1.24	1.41
C_4_AF + C_3_A	3.59	12.09	20.58	28.56	10.63	21.61	32.32	42.80

Samples with AM limited to 1.6 will formed less C_3_S as a result of the decreased consumption of CaCO_3_ in the raw meal. This effect is more evident at higher SFCC levels due to the lower calcium content. Consequently, the lower Ca/Si ratio led to an increasing C_2_S content^5^. Up to 15% SFCC could be co-processed to produce ordinary tricalcium C_3_S-based Portland clinker.

Constraining the AM stabilised Ca_2_(Al,Fe)_2_O_5_ due to increased Fe_2_O_3_ contents. In Table 5, C_4_AF corresponds to the sum of Ca(Al,Fe)_2_O_4_, Ca_2_(Al,Fe)_2_O_5_, and part of Ca_3_(Al,Fe)_2_O_6_^60^. Although no regulatory limits are specifically established on C_4_AF, the content of this phase usually ranges between 8 and 13% in OPC clinker^5^ and up to 22% for high-ferrite Portland cement (HFPC)^79^. Consequently, the co-processing of more than 15% of SFCC would be suitable for HFPC systems, in which properties such as high resistance to abrasion, sulfate and chloride attack are required, while also reducing CO_2_ emissions during clinkerisation^80,81^. By co-processing 8% SFCC in clinker, Lin et al*.*^30^ associated the low compressive strength with the high C_3_A content (19%). In the present study, although the modelling showed the C_3_A amount incorporated into the C_3_(A,F) content, the results indicated that this limit was not exceeded even for systems dosed with 15% SFCC. According to the European standard (EN 197-1), the maximum permissible limit of C_3_A in clinker is 9% for sulfate resisting pozzolanic cement (CEM IV)^82^. In contrast, the American standard (ASTM C150-07) allows this value to reach 15% of the clinker in the production of high early strength cement (type III)^83^. This implies that up to 10% of SFCC (S10F) could be co-processed, complying with European requirements, and up to 15% (S15F) according to American standards. The sum of C_4_AF and C_3_A increased when fixing the AM as more Fe_2_O_3_ was incorporated into the system. However, the values meet the usual thresholds (30%)^5,66,83^ for co-processing up to 10% SFCC. In addition to the C_3_A content, there are other determining factors on the clinker reactivity. The optimisation of sulfates in cement production can adjust hydration stages even with higher aluminate contents, delaying the C_3_A reaction and improving the hydration of C_3_S^84^. Thus, the poor reactivity of the SFCC clinker from previous studies could be associated with the low content of C_3_S in the sample and the adjustment of SO_3_ in the cement.

Ca_3_MgAl_4_O_10_ (C_3_A_2_M) is one of the first crystallisation products of Mg and Al from the melt phase during cooling^85,86^. No previous studies have reported the presence of C_3_A_2_M in experimental clinker samples, and there are some uncertainties about the stability of this phase as a solid in industrial clinker^63^. C_3_A_2_M was verified in the modelling of CaO-Al_2_O_3_-MgO systems^85,86^ and synthesised in laboratory scale studies^87,88^, with the hydration rate directly associated with its degree of crystallinity. High levels of SFCC lead to the growth of C_3_A_2_M due to the greater availability of alumina to react with MgO present within CaCO_3_ AP (0.29 wt%). Consequently, this behaviour can be associated with a decrease in free MgO. This drop is advantageous since the hydration of MgO is slow and may cause deleterious expansion of the hardened paste by forming Mg(OH)_2_^5^. The MgO contents are below the standardised limit (6.0%)^83^.

Na_2_Ca_8_Al_6_O_18_ is the orthorhombic tricalcium aluminate (C_3_A-o)^67,68^. Its content gradually reduced as the SFCC content increased and was not present when the AM was set to 1.6. Compared to the cubic form, C_3_A-o reacts faster with gypsum (CaSO_4_.2H_2_O) in cement, forming longer ettringite crystals and leading to problems related to setting time and workability^89^. Na_2_Ca_3_Al_16_O_28_ is a sodium-modified calcium aluminate. The effect on cement hydration is unknown. In a previous study it was predicted only at trace concentrations (< 0.05) via thermodynamic modelling of the CaO-Al_2_O_3_-NaO system^90^.

Table 5 reveals that there was a gradual decrease in the free CaO content with increasing SFCC. Thus, the residue improved the clinker burnability, reducing the percentage of uncombined CaO. This behaviour may be related to the increased iron oxide content in the raw meal^5^. Thermodynamic modelling shows that Ti from SFCC would be stabilised in Ca_3_Ti_2_O_7_. This phase is formed from the combination of perovskite (CaTiO_3_) and CaO, crystallising in its orthorhombic form^91^. Ca_3_Ti_2_O_7_ and other types of perovskite have already been verified in alumina-rich cement^92^. Their formation is associated with interacting with C_4_AF^93,94^. Unlike titanium, nickel remains in the clinker in its crystalline oxide form (NiO). Previous studies have shown that Ni added to a raw meal at up to 1.2% did not affect cement hydration reactions, nor did it pose an environmental risk through leaching^95,96^.

Simulation of cooling by the Scheil-Gulliver method determines the composition after the maximum conversion of the melt phase into solids during cooling^23^. The melt phase remaining after cooling corresponds to the non-crystalline clinker fraction^62^. Raising the SFCC content increased this non-crystalline fraction due to chemically non-combined residual elements (Ni, Ti, Na, La, V, P, K, S, Zn, and Mn). Nickel (Ni), titanium (Ti), and sodium (Na) exhibited the highest potential for combination with crystalline phases. On the other hand, lanthanum (La), vanadium (V), and phosphorus (P) were not detected in the solid fraction after cooling. The concentration of these elements in the remaining melt suggests their propensity to be distributed in the last phases to recrystallise during cooling, particularly in C_4_AF. This behaviour corroborates the findings of Harada et al.^97^, who reported the concentration of La_2_O_3_ (3% of the raw meal) in the interstitial regions of the clinker, primarily around the contours of C_3_S and C_2_S grains. Co-processing pure V_2_O_5_ at levels above 0.5% of the raw meal resulted in the formation of AlV_2_O_6_ and CaV_2_O_6_^98^. However, below these concentrations of La_2_O_3_ and V_2_O_5_, no crystalline compounds containing these elements were detected. This effect may explain the absence of crystalline phases containing these oxides in the modelled clinkers, as the maximum content of La_2_O_3_ and V_2_O_5_ in the raw meal (S20F) were 0.48% and 0.14%, respectively. Consequently, further studies are necessary to explore the impact of increasing these elements individually in the raw meal and to map their distribution in the clinker phases. Nonetheless, the results suggest that fixing the alumina modulus at 1.6 increased the potential for combining residual oxides into crystalline phases. This characteristic is essential for the environmentally safe production of clinker containing waste raw materials.

Compared to previous studies on Portland clinker containing SFCC^30,46^, the raw meal proportioning by thermodynamic modelling produced potentially more reactive clinkers with reduced limestone consumption and, therefore, lower CO_2_ emissions. In addition to the raw materials optimisation parameters, fixing the alumina modulus was essential to guarantee the C_3_A limitation. Samples with AM set at 1.6 have the potential to be environmentally more benign since higher levels of trace elements from the SFCC could be incorporated into the crystalline fraction of clinker. Furthermore, the dosed samples could produce clinkers with more than 50% C_3_S even when 15% SFCC is co-processed into the raw meal. Although the purity of the raw materials used with the SFCC may have contributed to the enhanced stabilisation of C_3_S in the modelled clinker, the data reported in this study suggest that the proposed dosing method can be applied to systems containing alternative sources, such as natural limestone and clays. In this sense, the proposed method represents a strategy to improve the clinker reactivity regardless of the purity of the raw materials employed.

### Considerations about the melt phase

Raw meal melting temperature is an important factor in the clinkering process. It is related to the melt phase formation and interferes with the hot meal stickiness on the inner coating of the kiln^5^. Melt control is relevant as it can lead to expensive issues for the furnace infrastructure. Higher levels of SFCC increase the Al_2_O_3_ and Fe_2_O_3_ contents in the raw meal. As a result, the melt phase, C_3_S formation, and melting point are reached at lower temperatures^3^. In this sense, the dosing of raw meals containing alumina-rich residual raw materials needs to guarantee the minimum requirements to avoid sample melting during clinkering. The fusibility test by heating microscopy allows simulation of industrial production and verification of the sample dimensional evolution during heating and cooling.

**Figure 4:**
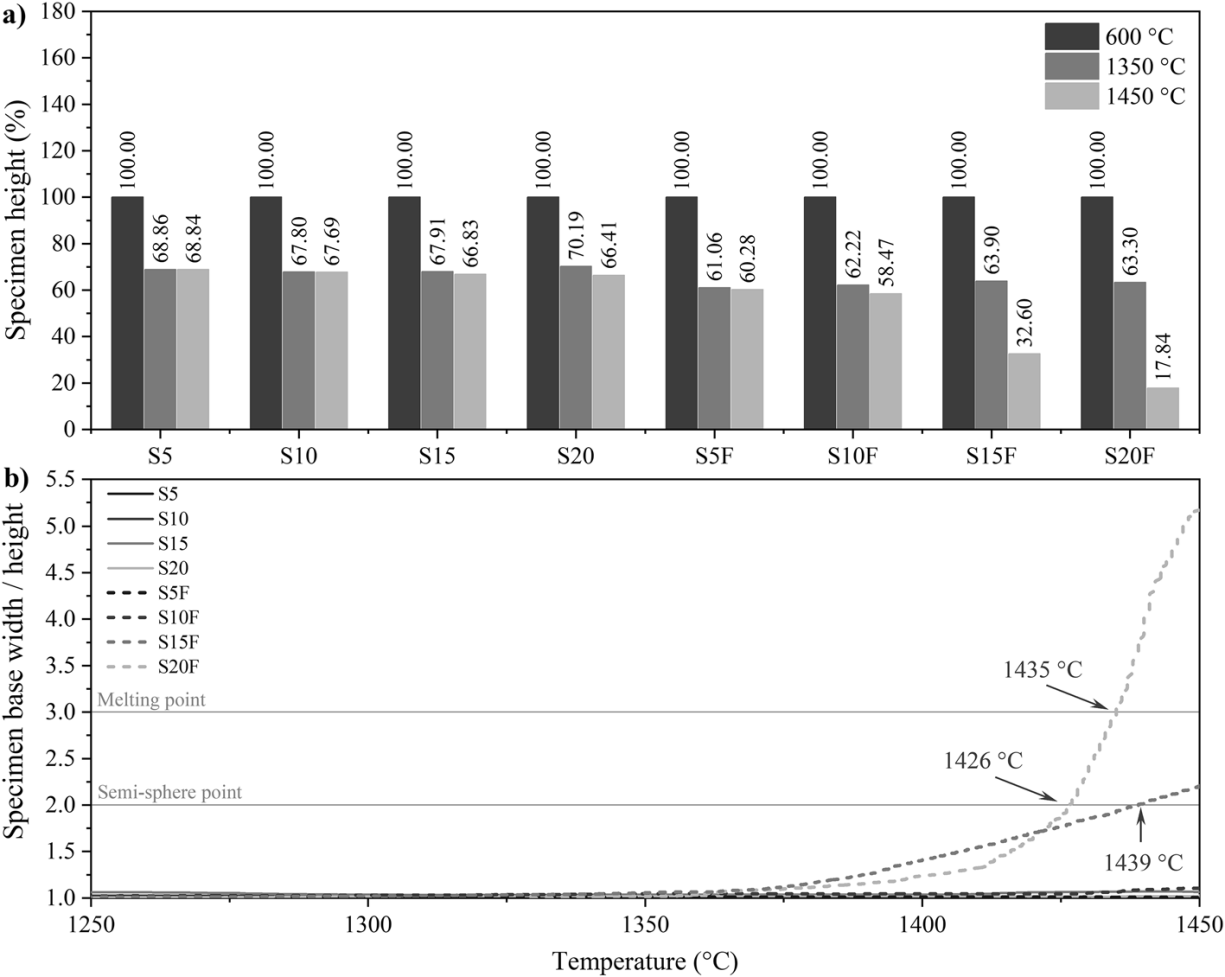
Sample properties during the fusibility test. (**a**) Specimen height at the start of decarbonation (600 °C), maximum silicate formation (1350 °C), and clinkering (1450 °C) temperatures. (**b**) Identification of semi-sphere and melting points between 1250 and 1450 °C.

Heating microscopy results are usually presented in terms of specimen height. Figure 4a shows the height at critical temperatures. Immediately before the CaCO_3_ decarbonation (600 °C), when the CO_2_ is volatilised, the sample loses between 30 and 40% of its height. At 1350 °C, the formation of C_3_S starts, and at 1450 °C, the clinkerisation is complete. The difference between the height at these last two temperatures is commonly used as an indication of the melting point. However, it can be inaccurate because other factors can lead to sample shrinkage. An example is the decomposition of phases and volatilisation of components between 1350 and 1450 °C. The DIN 51730^69^ standard classifies the half-sphere and melting points as the temperature at which the specimen base width and height ratio reaches 2 and 3, respectively. Figure 4b presents the evolution of this ratio for the dosed samples. It confirms that only the S15F and S20F clinkers started the melting process.

**Figure 5:**
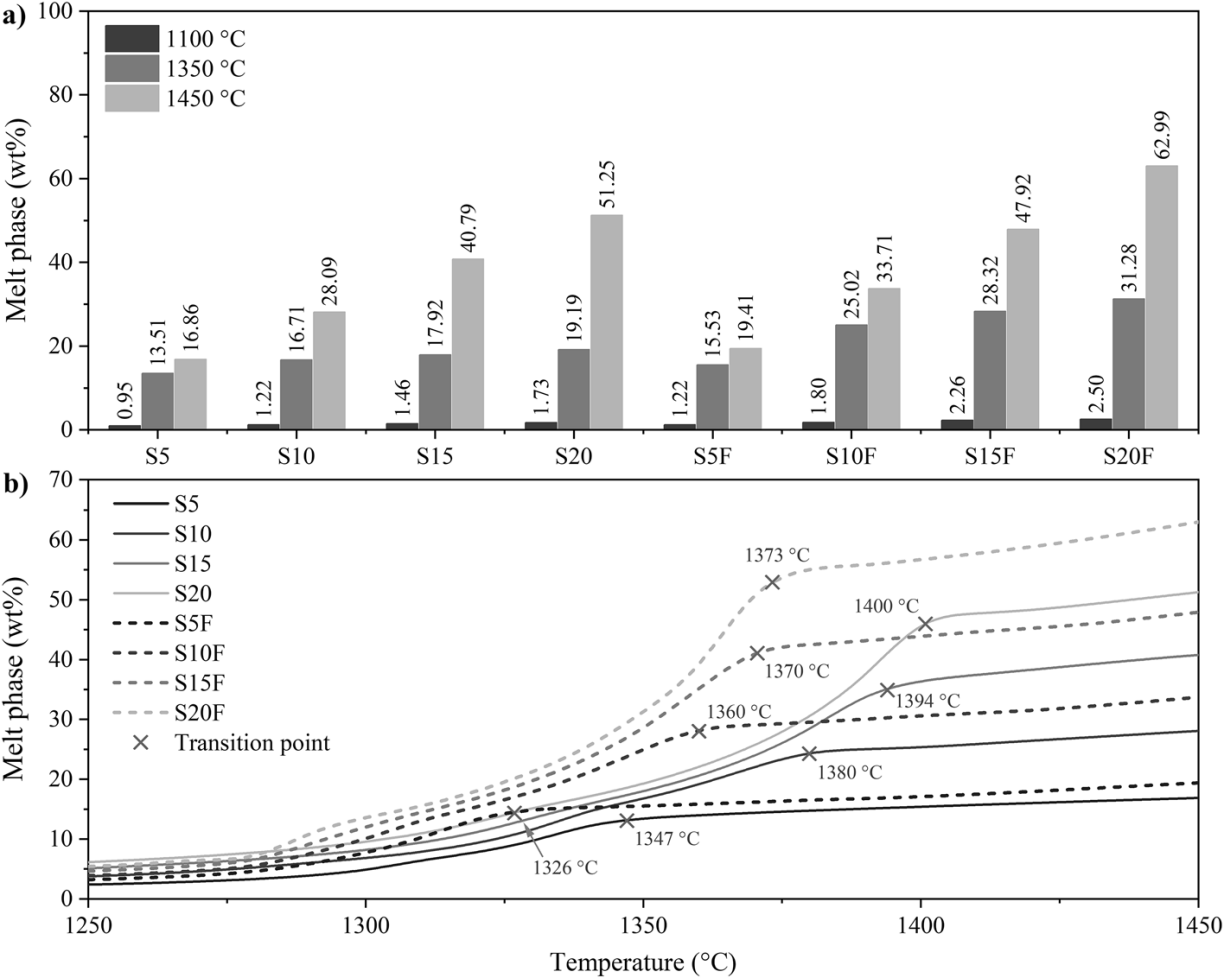
Melt phase content determined by thermodynamic modelling. (**a**) After decarbonation (1100 °C), maximum silicate formation (1350 °C), and maximum clinkering (1450 °C) temperatures. (**b**) Phase evolution between 1250 and 1450 °C.

The melt phase content has already been associated with sample melting during clinkering^5^. Thus, it would be expected that the molten samples would present the highest melt phase contents among the modelled systems (Fig. 5a). However, sample S20 has a melt phase content between that of S15F and S20F yet maintained its shape during the heating microscopy analysis. The correlation between the melt phase viscosity and the softening effect of the clinker nodule can explain this behaviour. Previous studies reported a direct relationship between the decrease in melt phase viscosity during clinkerisation and the increase in deformation at high temperatures, implying a higher penetration rate into the pores of the refractory lining of industrial kilns^16,63^. The temperature, variations in AM, and the presence of minor elements govern the evolution of the melt phase viscosity^16^. Specifically, fluorides, chlorides, alkaline sulfates, and MgO are associated with decreased viscosity, while alkalis and phosphorus promote the opposite effect^16,99^.

Figure 5b shows the evolution of the melt content in the region close to the clinkering temperature. Two main zones characterise the curve. At first, there is growth in the melt content, reaching at least 70% of the maximum melt phase content. This is followed by a second zone where the melt phase content tends to stabilise, varying less than 30% over the phase formation^3,10^. The boundary between zones indicates the onset of C_3_S formation. The distribution of these points agrees with the trend expected by changes in alumina modulus, which specify that lower AM improves the raw meal burnability, reducing the temperature of C_3_S formation^5,66^. Therefore, the inflection in the melt phase formation rate occurs at lower temperatures in the samples with AM limited to 1.6 (Fig. 5b). The temperatures also differ due to other factors interfering in the process. In these samples, the main factor is the Fe_2_O_3_ content, which acts as a flux, increasing the percentage of melt phase^66^.

**Figure 6:**
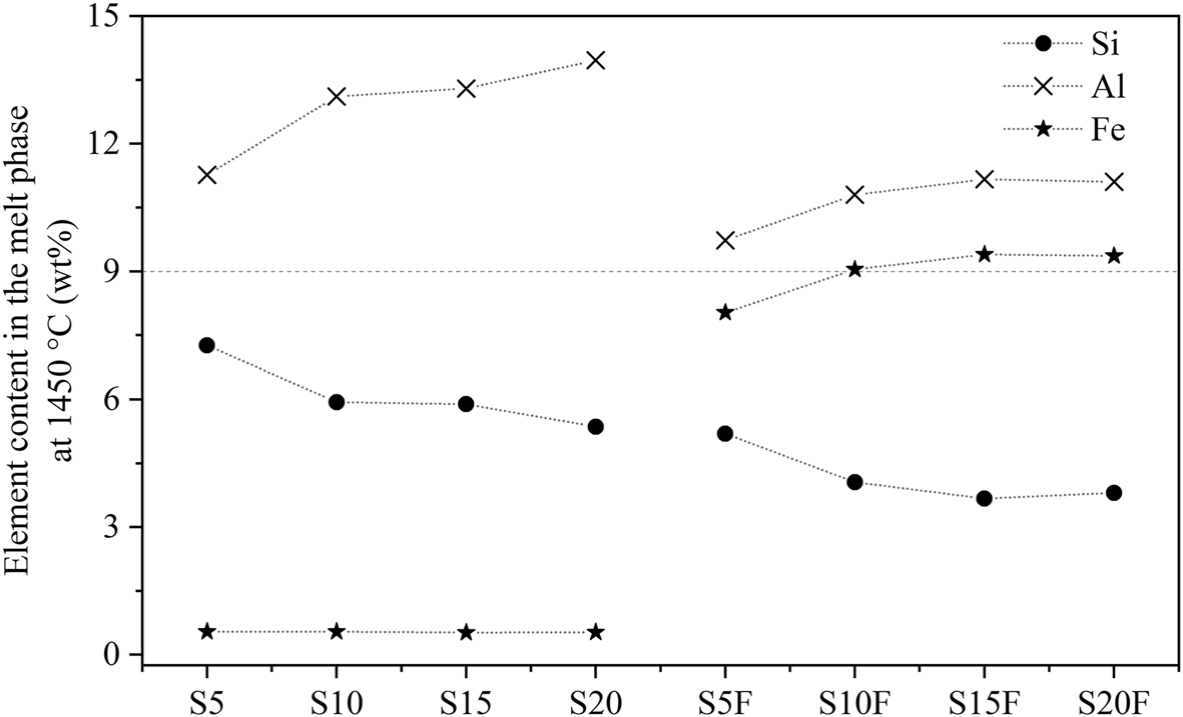
Si, Al, and Fe content in the melt phase at 1450 °C determined by thermodynamic modelling. Elemental weight fraction in relation to the melt phase content.

In the zone of stabilisation of the melt phase content, the viscosity of the melt phase varies significantly as a function of temperature. The melt content is crucial for the reaction of CaO, which gradually dissolves in the melt phase^5^. Lower viscosities improve CaO mobility and the diffusion rate, minimising reaction time and temperature for C_3_S crystallisation^100^. Iron is a mineraliser that increases the content and reduces the viscosity of the melt phase^5^. The opposite is true for Si^66^. Figure 6 shows the Si, Al, and Fe contents expected in the melt phase at the maximum clinkering temperature. The melt viscosity is a function of the atomic size of the elements and the forces between them^66^. Increasing the SFCC content resulted in a higher aluminium concentration in the melt phase. Although SFCC is composed of aluminosilicates, the silicon content in the melt phase differed from the Al tendency. This disparity is attributed to the dosing optimisation, which reduced the demand for SiO_2_ AP to maximise C_3_S (Tables 3 and 4). For the optimised samples (AM fixed at 1.6), the availability of Al and Si in the melt phase decreased while the iron content increased. Among these samples, systems S15F and S20F started the melting process (Fig. 4), suggesting that the melt phase viscosity should be increased to minimise deformation at high temperatures. Thus, the maximum feasible Fe concentration in the melt phase was 9% (S10F), as this element contributes to viscosity reduction and an increase in the melt phase content^5^. In this scenario, reducing the viscosity improves the nucleation and crystallisation reactions of C_3_S in C_2_S, optimising the clinker properties and lowering the production temperature.

Raw meal fusibility is a crucial factor in the production of industrial clinker. Melting of the sample during heat treatment can cause irreversible damage to the furnaces. The main concern is the melting of the material throughout the process and the consequent adherence to the refractory coating. The results suggest that the Fe content should not exceed 9% so that a minimum viscosity and CaO diffusion rate are guaranteed.

In general, the melt phase content of conventional Portland cement (CEM I) clinker at the maximum production temperature is around 22%^3^. It should be considered, however, that this reference percentage was estimated based on simplified equations, such as those of Dahl^101^ and Lea^5^. Compared to thermodynamic modelling, these methods tend to underestimate the melt phase content since they neglect the effect of minor elements acting as fluxes. The association between modelling and the fusibility test demonstrates that the samples tend to melt when they exceed 40% melt phase, and Fe constitutes more than 9% of this phase. Thus, limiting these parameters can be a criterion to reduce the risk of melting the mixture during clinkering.

### Considerations about the cooling

**Figure 7:**
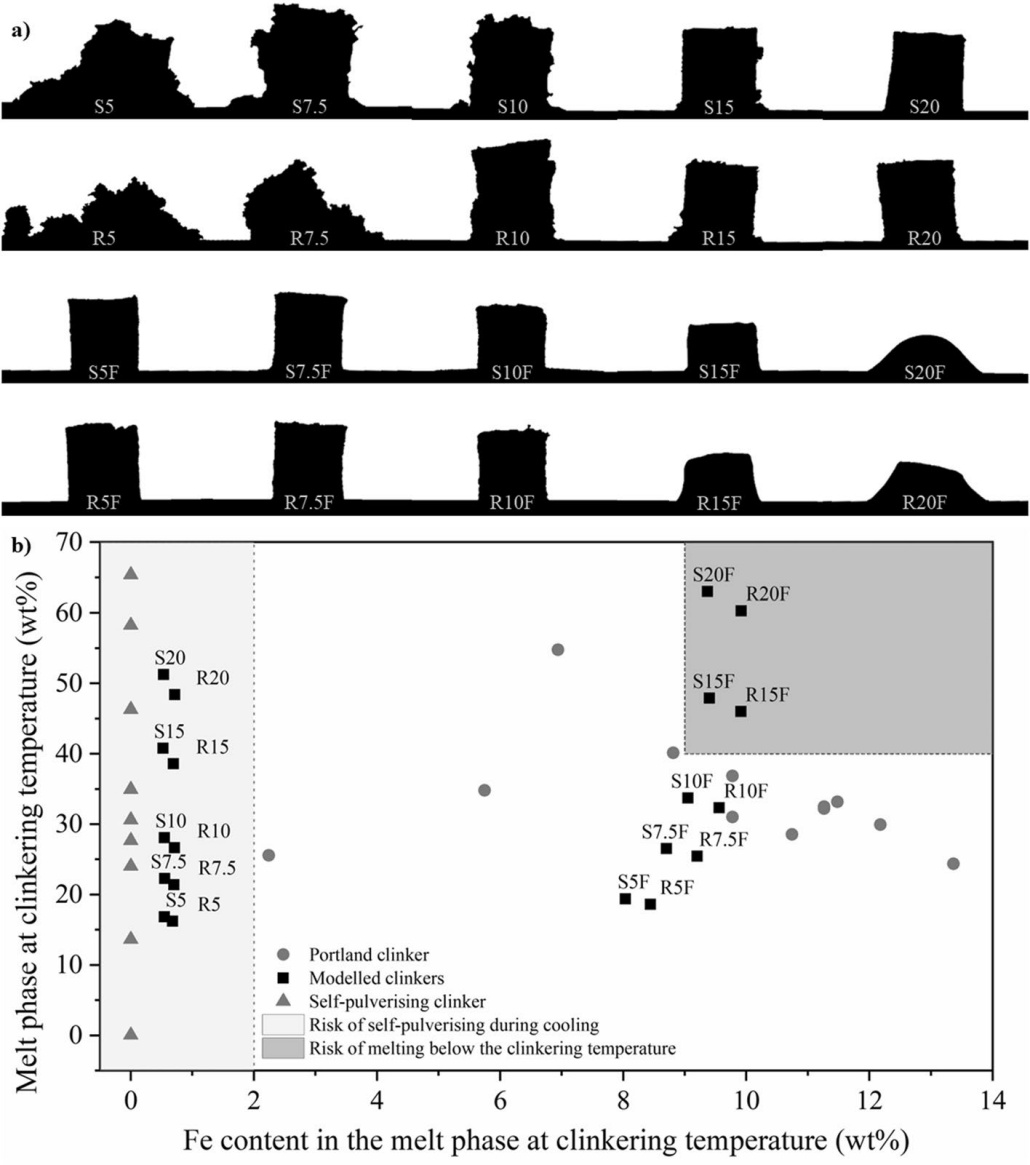
Parameters for predicting sample melting below the clinkering temperature or collapse during cooling. (**a**) Shape of the samples after the fusibility test up to 1450 °C followed by cooling to 200 °C. (**b**) Relation between the iron content in the melt phase amount at 1450 °C by thermodynamic modelling. Validation of risk zones based on self-pulverising and Portland clinkers from the literature^23,31,70–75^.

The heating microscopy fusibility test analyses the sample up to the production temperature. However, analysis during cooling is also an essential factor in studying clinker. After reaching the maximum melt phase content, the sample is rapidly cooled during manufacturing. This process guarantees C_3_S stability, which will not be reconverted into C_2_S and CaO. In addition, the melt phase is solidified mainly in C_3_A and C_4_AF, with minor contents of C_3_S, C_2_S, and others^66^. During cooling, the sample may change shape and volume. Figure 7a shows the clinkers after heating microscopy up to 1450 °C and subsequent cooling to 200 °C.

Samples from the first dosing step (AM > 27) self-pulverised. This is desired for some types of cement to reduce costs and CO_2_ emissions associated with clinker grinding. This property is usually obtained by changing the volume of the phases during cooling, either by forming new compounds or stabilising higher-volume polymorphs^71^. The thermodynamic modelling did not reveal the formation of recognizably expansive phases (Table 5), such as γ-dicalcium silicate (γ-Ca_2_SiO_4_) or rankinite (Ca_3_Si_2_O_7_)^102^. These phases are commonly promoted for self-pulverisation and the software database covers their formation^60^. However, it should be considered that the high viscosity of the molten fraction interferes with the ionic mobility and the stabilisation of silicates during cooling.

Figure 7 also shows a set of reference samples prepared with analytical grade reactants (R5, R7.5, R10, R15, R20, R5F, R7.5F, R10F, R15F, and R20F), aiming to isolate the effect of SFCC impurities (La, V, Ni, Ti, Na, and P). However, the behaviour of references and SFCC clinkers were essentially identical. It demonstrates that pulverisation is associated with a common factor among the samples. The pulverisation of samples with free AM still needs to be better understood and may be related to changes in viscosity.

**Figure 8:**
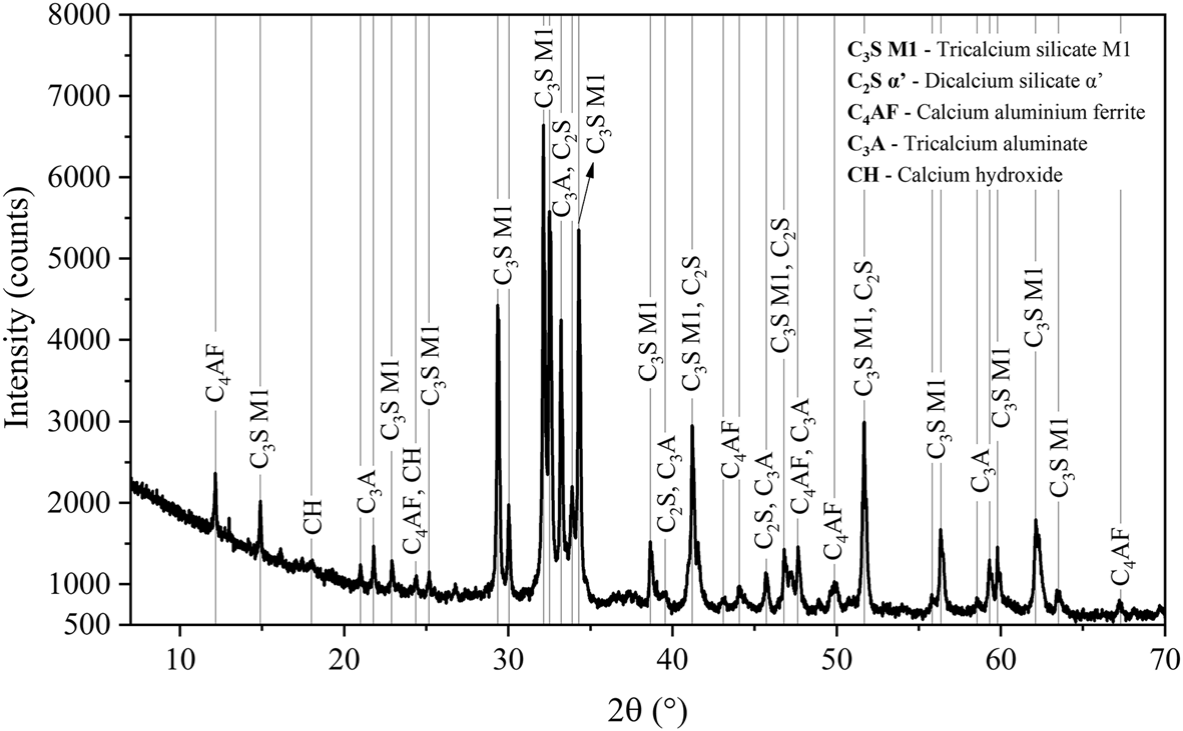
X-ray diffractogram of clinker S10F obtained by XRD/Rietveld. C_3_S M1: tricalcium silicate M1 (Ca_3_SiO_5_); C_2_S α’: dicalcium silicate α’ (ICSD 81097, Ca_2_SiO_4_); C_4_AF: calcium aluminium ferrite (ICSD 9197, Ca_2_(AlFe)O_5_); C_3_A: tricalcium aluminate (ICSD 1841, Ca_9_(Al_2_O_6_)_3_); CH: calcium hydroxide (ICSD 15471, Ca(OH)_2_).

The phase composition of modelled clinkers can be verified by experimental techniques such as quantitative XRD analysis utilising the Rietveld method. As an example, Fig. 8 presents an experimental validation of the composition of clinker produced with 10% SFCC and AM set at 1.6. XRD/Rietveld analysis revealed the formation of tricalcium silicate with a monoclinic structure instead of the rhombohedral (Table 6). C_3_S M1, the most stable polymorph at room temperature, is typically found in industrial-scale clinkers^5^. The alteration in the crystalline structure can be attributed to the presence of minor elements in the raw meal, which was not captured by the clinkerisation modelling due to the limited consideration of polymorphs other than C_3_S R in the utilised database^60^. It is worth noting that the experimental analysis indicated a higher C_3_S content. This difference was compensated by the decrease in C_2_S amount, which was only observed in its α’ polymorph, corroborating the modelled results.

**Table 6:** Comparison between the composition of S10F clinker predicted by thermodynamic modelling and quantified by X-ray diffractometry combined with the Rietveld method (XRD/Rietveld). Values in parentheses represent the estimated error.

Composition (wt%)	Modelling	XRD/rietveld
Ca_3_SiO_5_ R	67.84	–
Ca_3_SiO_5_ M1	–	73.47 (0.24)
Ca_2_SiO_4_ α’	6.49	3.71 (0.20)
Ca(Al,Fe)_2_O_4_	0.37	–
Ca_2_(Al,Fe)_2_O_5_	10.90	8.19 (0.11)
Ca_3_(Al,Fe)_2_O_6_	10.34	13.08 (0.12)
Ca_3_MgAl_4_O_10_	1.97	–
Na_2_Ca_3_Al_16_O_28_	0.04	–
CaO	0.57	–
MgO	0.16	–
NiO	0.16	–
Ca_3_Ti_2_O_7_	0.16	–
Melt	0.97	–
Ca(OH)_2_	–	1.55 (0.06)
C_4_AF + C_3_A	21.61	21.27
Rwp (%)	–	5.59

The absence of modelled minor phases (such as Ca_3_MgAl_4_O_10_, Na_2_Ca_3_Al_16_O_28_, CaO, MgO, NiO, and Ca_3_Ti_2_O_7_) may have contributed to the observed increase in C_3_S quantification. The experimental results were in agreement with the thermodynamic modelling, particularly in terms of the combined content of C_4_AF and C_3_A, corresponding to approximately 21% of the clinker. The experimental analysis also showed approximately 1.5% of calcium hydroxide, which may be related to partial hydration of the clinker during sample handling and experiment execution. For example, CaO is a highly reactive compound and hydrates when exposed to environments with relative humidity exceeding 10%^103^.

Although the results do not allow for defining the cause of self-pulverisation of the samples in which AM varied freely, the existence of higher-risk areas is evident in Fig. 7b. The boundary conditions determined in the previous dosage steps delimited the risk areas of self-pulverisation during cooling and melting during clinkering. The delimitation of these zones was validated through modelling experimental data of self-pulverising^71^ and Portland^23,31,70,72–75^ clinkers from previous studies.

The Fe content in the melt fraction interferes with the viscosity and compounds mobility, increasing the risk of self-pulverisation on cooling when below 2% and of melting below the clinkering temperature when above 9%. However, the limit of 9% Fe in the melt phase only promoted the melting of the sample when associated with a melt phase content above 40%.

### Method framework

**Figure 9:**
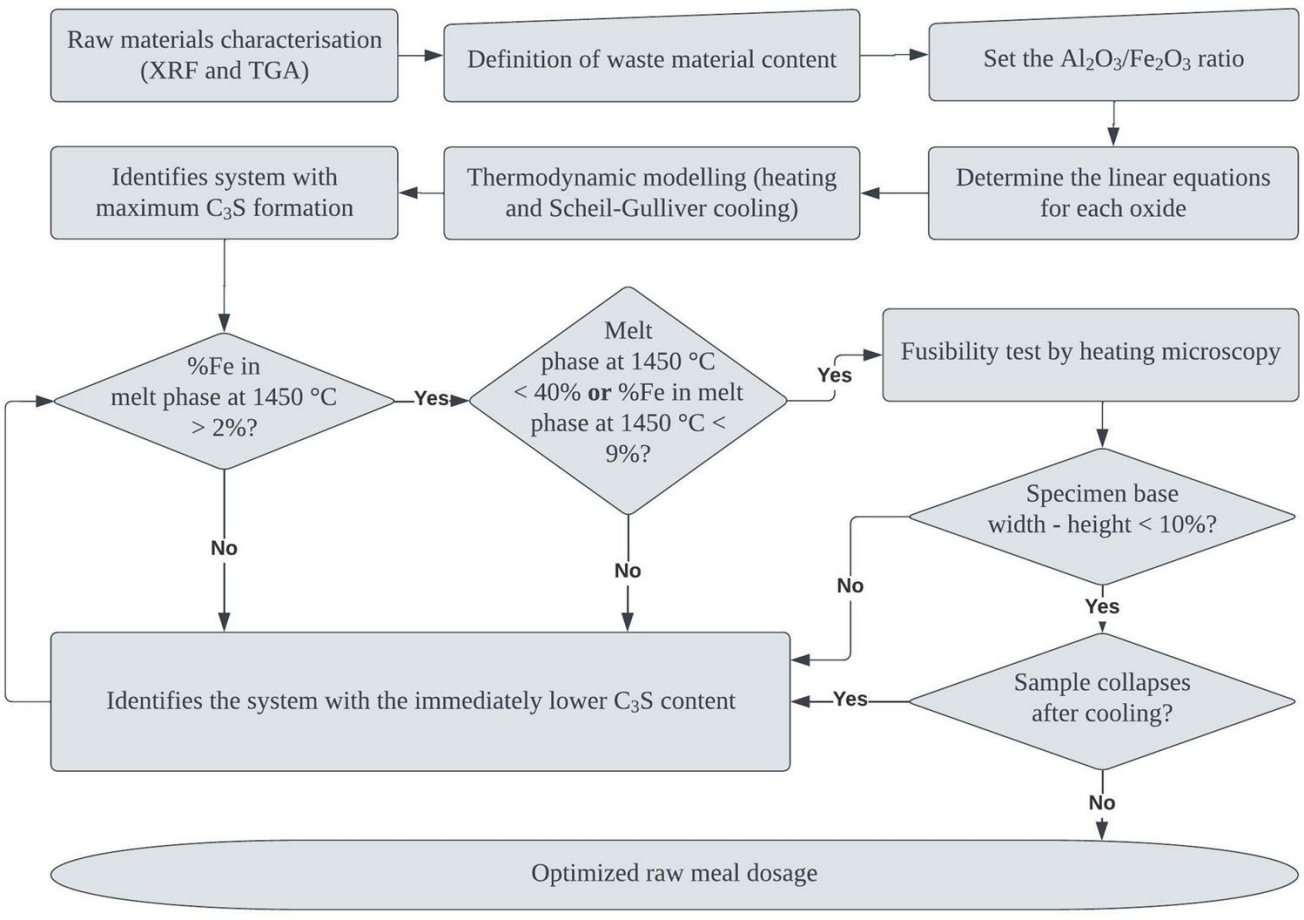
Flowchart of the raw meal proportioning method utilising thermodynamic modelling and co-processing aluminous alternative materials.

Figure 9 summarises the flowchart of the raw meal proportioning method developed in this study. The steps were formulated according to the SFCC case study, but the framework can be applied to other alumina-rich alternative materials. The application to other systems is appropriate since the factors are considered as a function of their composition. The framework delimitation for co-processing alumina-rich materials occurred due to the AM fixation step. The utilisation of materials based on other elements may have different implications on chemical moduli and phase formation. In this sense, further investigations can be conducted to define the appropriate calculation approach considering the chemical characteristics of other types of raw materials. As shown in Fig. 7, the delimited parameters corroborate experimental samples from the literature. The validation system was limited due to the availability of peer-reviewed publications detailing complete information on the chemical and mineralogical composition of the raw meals and temperature evolution during clinkering.

The method starts with the chemical characterisation of the raw materials, followed by determining the content of co-processed alternative feedstock. In industry, this definition can be based on the volume of cement production and the amount of alternative feedstock generated within a reasonable distance from the plant. The alumina modulus is then defined, and the input equations are determined according to Eqs. (2 and 3) of this study. Then the thermodynamic modelling simulates the heating and cooling of the system. The outputs are filtered based on the C_3_S content, amount of melt phase, and iron content in the melt at the maximum clinkering temperature. The optimised composition is then evaluated by heating microscopy to certify that the sample will not reach the melting point before clinkering and will not pulverise during cooling. In this way, the method allows for defining a raw meal optimised composition, maximising the content of co-processed residue in clinker production, and guaranteeing cement reactivity.

The main limitations of the method are associated with the technique applied to determine the residue oxide composition. In this study, XRF was used with a wide range of oxides and in a pure material pressed pellet. However, several factors can contribute to a decrease in the precision of chemical analysis, including inadequate specimen preparation, the collection of non-representative samples, variations in preparation techniques (powder, pressed pellet, fused, etc.), the degree of dilution, the detection limit, and challenges related to equipment calibration^104^. These systematic errors introduce experimental uncertainties that can potentially lead to inaccurate estimation of residue impurities, compromising the accuracy of predictive modelling.

A second limiting factor is the modelling software database. The set of available phases met the demands of the method for Portland clinker, but the system could be improved when considering the formation of C_3_S and β-C_2_S polymorphs. For the coprocessing of aluminous waste, it would be relevant to model systems for dosing calcium sulfoaluminate cement (CSA cements). This is still unfeasible on FactSage because the databases need to consider the formation of essential phases in this cement, such as ye'elimite.

Future studies may explore essential questions about the effects of iron, seeking a better understanding of the stabilisation of calcium aluminium ferrite solutions and changes in melt phase viscosity and phase volume over the course of simulations. It is important to emphasise that thermodynamic modelling is not intended to replace experimental techniques but to act as an additional tool, optimising decisions and concentrating efforts on relevant issues. These answers can bring important solutions to the manufacturing process and optimise the production of more sustainable cements.

## Conclusions

The proposed method optimised the composition of the raw meal, giving clinkers with more C_3_S (potentially more reactive) and limiting the C_3_A content in compliance with the European EN 197-1 (9%) and American ASTM C150-07 (15%) standards. Limestone consumption was lower compared to previous studies on Portland clinker containing SFCC, reducing CO_2_ emissions related to material decomposition.

The dosed samples could produce clinkers with more than 50% C_3_S even when 15% SFCC was co-processed into the raw meal. Thermodynamic modelling allowed dosing clinkers with a higher content of chemically combined SFCC. This characteristic is essential for the environmentally safe production of clinker containing alternative feedstocks.

Considering modelling results and the fusibility test it is demonstrated that clinkers tend to melt when they exceed 40% melt phase, and Fe constitutes more than 9% of this phase. Thus, limiting these parameters can be a criterion to reduce the risk of melting the mixture during clinkering, reducing maintenance costs, and improving the life expectancy of industrial kilns.

Although the sample self-pulverisation mechanism is still not completely understood, this behaviour seems related to high viscosities and Fe contents below 2% in the melt phase at clinkering temperature (1450 °C).

Optimising the chemical combination of impurities elements, maximising cement reactivity, reducing consumption of natural limestone and clay, and proper disposal of SFCC are positive implications of the method adopted for clinker production. This approach demonstrates the great potential for production of more sustainable cements while minimising the need of using natural resources.

### Supplementary Information

Supplementary Information.

## Data Availability

The data associated with this paper are openly available from the University of Leeds Data Repository, at https://doi.org/10.5518/1394.
