# Supplementary material for: Thermodynamic modelling of cements clinkering process as a tool for optimising the proportioning of raw meals containing alternative materials
Source: Sci Rep. 2023 Oct 16;13:17589. doi: 10.1038/s41598-023-44078-7 (PMC10579411; doi:10.1038/s41598-023-44078-7)
Supplement: Supplementary file 1 — Supplementary Information. [file 41598_2023_44078_MOESM1_ESM.pdf]

Thermodynamic modelling of cements clinkering process as a tool for optimising the proportioning of raw meals containing alternative materials

Ana R. D. Costa, Mateus V. Coppe, Wagner V. Bielefeldt, Susan A. Bernal, Leon Black, Ana P. Kirchheim, and Jardel P. Gonçalves

Supplementary information

Example of determining the equations for modelling

Table S1 shows a simplified example of the oxide composition of the raw materials to illustrate the calculation of the input equations for the dosing method by thermodynamic modelling. The example considers co-processing 10 wt.% SFCC and the alumina modulus set to 1.6.

Oxides (%)	SFCC	$CaCO_3$ AP	SiO_2 AP	Fe_2O_3 AP
SiO_2	55.00	1.00	100.00	0.00
Al_2O_3	44.00	1.00	0.00	1.00
Fe_2O_3	1.00	0.00	0.00	98.00
CaO	0.00	56.00	0.00	1.00
CO_2	0.00	42.00	0.00	0.00

Table S1. Oxide composition of the raw materials for the simplified example.

The determination of the fixed Fe_2O_3 AP content is shown in equation S1.

$$F_{fixed} = \frac{10 \cdot 44.00}{1.6 \cdot 98.00} = 2.81\% \quad \text{(S1)}$$

The angular coefficients for the input equations are calculated as in equation S2.

$$\begin{bmatrix} A_{SiO2} \\ A_{Al2O3} \\ A_{Fe2O3} \\ A_{CaO} \\ A_{CO2} \end{bmatrix} = \begin{bmatrix} 0.55 & 0.01 & 1.00 & 0.00 \\ 0.44 & 0.01 & 0.00 & 0.01 \\ 0.01 & 0.00 & 0.00 & 0.98 \\ 0.00 & 0.56 & 0.00 & 0.01 \\ 0.00 & 0.42 & 0.00 & 0.00 \end{bmatrix} \cdot \begin{bmatrix} 0 \\ -1 \\ 1 \\ 0 \end{bmatrix} = \begin{bmatrix} 0.99 \\ -0.01 \\ 0.00 \\ -0.56 \\ -0.42 \end{bmatrix} \quad \text{(S2)}$$

The linear coefficient for each oxide is calculated from Equation S3. Where Sfixed was the fixed SFCC content for the system (10 wt.%), Cmax was the maximum $CaCO_3$ AP content in the raw meal (100 - Sfixed – Ffixed = 87.19), Smin was the minimum content of SiO_2 AP (0 wt.%), and Ffixed was the content of Fe_2O_3 AP fixed in the system (2.81 wt.%).

$$\begin{bmatrix} B_{SiO2} \\ B_{Al2O3} \\ B_{Fe2O3} \\ B_{CaO} \\ B_{CO2} \end{bmatrix} = \begin{bmatrix} 0.55 & 0.01 & 1.00 & 0.00 \\ 0.44 & 0.01 & 0.00 & 0.01 \\ 0.01 & 0.00 & 0.00 & 0.98 \\ 0.00 & 0.56 & 0.00 & 0.01 \\ 0.00 & 0.42 & 0.00 & 0.00 \end{bmatrix} \cdot \begin{bmatrix} 10.00 \\ 87.19 \\ 0.00 \\ 2.81 \end{bmatrix} = \begin{bmatrix} 6.37 \\ 5.30 \\ 2.85 \\ 48.85 \\ 36.62 \end{bmatrix} \quad \text{(S3)}$$

In this context, the linear equations applied as inputs in FactSage would be indicated below (S4 to S8). The variable *x* (SiO_2 AP content) would be configured in the software to go through values from 0 to 22.5% with a step of 0.5, covering 46 scenarios (raw meal compositions).

$$Y_{SiO2}=6.37+0.99x \quad \text{(S4)}$$

$$Y_{Al2O3}=5.30-0.01x \quad \text{(S5)}$$

$$Y_{Fe2O3}=2.85 \quad \text{(S6)}$$

$$Y_{CaO}=48.85-0.56x \quad \text{(S7)}$$

$$Y_{CO2}=36.62-0.42x \quad \text{(S8)}$$

Oxide composition of modelled systems

Table S2 details the oxide composition of the clinker raw meals dosed in this study.

Sample	CaO	SiO_2	Fe_2O_3	Al_2O_3	MgO	SO_3	P_2O_5	Na_2O	ZnO	K_2O	NiO	TiO_2	MnO	CO_2
S5	46.02	15.99	0.09	2.33	0.25	0.06	0.08	0.13	0.00	0.01	0.05	0.03	0.00	34.96
S7.5	45.74	15.30	0.11	3.44	0.25	0.06	0.09	0.13	0.00	0.02	0.07	0.05	0.00	34.74
S10	45.46	14.60	0.14	4.55	0.25	0.06	0.10	0.13	0.00	0.02	0.10	0.06	0.01	34.53
S15	44.61	13.71	0.20	6.77	0.24	0.05	0.12	0.14	0.00	0.03	0.14	0.09	0.01	33.89
S20	44.05	12.32	0.26	8.99	0.24	0.05	0.14	0.14	0.00	0.04	0.19	0.11	0.01	33.46
R5	46.00	15.98	0.09	2.33	0.25	0.07	0.06	0.13	0.00	0.01	0.00	0.00	0.00	34.95
R7.5	45.78	15.32	0.14	3.47	0.25	0.07	0.05	0.13	0.00	0.01	0.00	0.00	0.00	34.78
R10	45.44	14.60	0.15	4.55	0.24	0.06	0.05	0.13	0.00	0.01	0.00	0.00	0.00	34.53
R15	44.60	13.71	0.20	6.77	0.24	0.06	0.05	0.13	0.00	0.01	0.00	0.00	0.00	33.88
R20	44.04	12.32	0.26	8.99	0.24	0.06	0.05	0.13	0.00	0.01	0.00	0.00	0.00	33.46
S5F	45.73	15.12	1.45	2.33	0.25	0.07	0.08	0.12	0.00	0.01	0.05	0.03	0.01	34.74
S7.5F	45.17	14.23	2.17	3.44	0.24	0.07	0.09	0.13	0.00	0.02	0.07	0.04	0.01	34.32
S10F	44.61	13.35	2.88	4.55	0.24	0.07	0.10	0.13	0.00	0.02	0.10	0.06	0.01	33.89
S15F	43.49	11.60	4.30	6.76	0.24	0.07	0.12	0.13	0.00	0.03	0.14	0.09	0.01	33.03
S20F	42.06	10.36	5.73	8.98	0.23	0.07	0.14	0.13	0.01	0.04	0.19	0.11	0.02	31.94
R5F	45.76	15.14	1.47	2.35	0.25	0.07	0.06	0.12	0.00	0.01	0.00	0.00	0.01	34.77
R7.5F	45.22	14.26	2.19	3.46	0.24	0.07	0.05	0.12	0.00	0.01	0.00	0.00	0.01	34.35
R10F	44.67	13.39	2.91	4.58	0.24	0.07	0.05	0.12	0.00	0.01	0.00	0.00	0.01	33.94
R15F	43.57	11.65	4.35	6.81	0.23	0.08	0.05	0.12	0.00	0.01	0.00	0.00	0.01	33.10
R20F	42.18	10.42	5.80	9.05	0.23	0.08	0.05	0.12	0.00	0.01	0.00	0.00	0.01	32.04

Table S2. Oxide composition of the clinker raw meals optimised by the proposed dosing method.

Table S3 presents the oxide composition of clinker raw meals from previous studies simulated for method validation.

ID	CaO	SiO_2	Al_2O_3	Fe_2O_3	K_2O	Na_2O	MgO	TiO_2	SrO	SO_3	P_2O_5	V_2O_5	Cr_2O_3	MnO	ZnO	Cl	F	T (°C)	Ref
01	58.16	16.17	5.05	5.39	1.04	0.65	8.08	0.25	0.00	0.85	3.25	0.04	0.08	0.42	0.04	0.03	0.51	1380	[31]
02	64.75	21.37	4.31	5.42	0.64	0.32	2.87	0.00	0.00	0.32	0.00	0.00	0.00	0.00	0.00	0.00	0.00	1450	[73]
03	68.91	24.48	3.22	0.82	0.39	0.67	1.51	0.00	0.00	0.00	0.00	0.00	0.00	0.00	0.00	0.00	0.00	1500	[72]
04	65.29	24.28	4.90	4.38	0.32	0.01	0.73	0.00	0.00	0.08	0.00	0.00	0.00	0.00	0.00	0.00	0.00	1470	[70]
05	65.50	23.75	4.80	4.32	0.31	0.14	0.87	0.00	0.00	0.30	0.00	0.00	0.00	0.00	0.00	0.00	0.00	1470	[70]
06	65.68	20.82	4.44	2.79	1.29	0.21	2.77	0.23	0.00	1.64	0.03	0.00	0.00	0.00	0.02	0.09	0.00	1500	[23]
07	66.56	21.25	6.34	5.23	0.27	0.13	0.07	0.01	0.09	0.01	0.04	0.00	0.00	0.00	0.00	0.00	0.00	1450	[74]
08	61.74	24.56	6.23	5.13	2.01	0.12	0.06	0.01	0.09	0.01	0.04	0.00	0.00	0.00	0.00	0.00	0.00	1360	[74]
09	62.59	24.90	6.31	5.21	0.27	0.50	0.07	0.01	0.09	0.01	0.04	0.00	0.00	0.00	0.00	0.00	0.00	1360	[74]
10	62.81	25.00	6.34	5.23	0.27	0.13	0.07	0.01	0.09	0.01	0.04	0.00	0.00	0.00	0.00	0.00	0.00	1360	[75]
11	62.35	24.81	6.29	5.19	1.01	0.13	0.07	0.01	0.09	0.01	0.04	0.00	0.00	0.00	0.00	0.00	0.00	1360	[75]
12	61.97	24.65	6.25	5.15	0.26	1.50	0.07	0.01	0.09	0.01	0.04	0.00	0.00	0.00	0.00	0.00	0.00	1360	[75]
13	53.90	36.10	10.00	0.00	0.00	0.00	0.00	0.00	0.00	0.00	0.00	0.00	0.00	0.00	0.00	0.00	0.00	1400	[71]
14	47.90	32.10	20.00	0.00	0.00	0.00	0.00	0.00	0.00	0.00	0.00	0.00	0.00	0.00	0.00	0.00	0.00	1400	[71]
15	56.40	33.60	10.00	0.00	0.00	0.00	0.00	0.00	0.00	0.00	0.00	0.00	0.00	0.00	0.00	0.00	0.00	1400	[71]
16	50.10	29.90	20.00	0.00	0.00	0.00	0.00	0.00	0.00	0.00	0.00	0.00	0.00	0.00	0.00	0.00	0.00	1400	[71]
17	58.60	31.40	10.00	0.00	0.00	0.00	0.00	0.00	0.00	0.00	0.00	0.00	0.00	0.00	0.00	0.00	0.00	1400	[71]
18	62.20	27.80	10.00	0.00	0.00	0.00	0.00	0.00	0.00	0.00	0.00	0.00	0.00	0.00	0.00	0.00	0.00	1400	[71]
19	56.70	23.30	20.00	0.00	0.00	0.00	0.00	0.00	0.00	0.00	0.00	0.00	0.00	0.00	0.00	0.00	0.00	1400	[71]
20	57.90	22.10	20.00	0.00	0.00	0.00	0.00	0.00	0.00	0.00	0.00	0.00	0.00	0.00	0.00	0.00	0.00	1400	[71]
21	58.90	21.10	20.00	0.00	0.00	0.00	0.00	0.00	0.00	0.00	0.00	0.00	0.00	0.00	0.00	0.00	0.00	1400	[71]

Table S3. Oxide composition of clinker raw meals simulated for method validation.
